# Photosynthesis, Light Use Efficiency, and Yield of Reduced-Chlorophyll Soybean Mutants in Field Conditions

**DOI:** 10.3389/fpls.2017.00549

**Published:** 2017-04-18

**Authors:** Rebecca A. Slattery, Andy VanLoocke, Carl J. Bernacchi, Xin-Guang Zhu, Donald R. Ort

**Affiliations:** ^1^Department of Plant Biology, University of Illinois at Urbana-Champaign, UrbanaIL, USA; ^2^Carl R. Woese Institute for Genomic Biology, University of Illinois at Urbana-Champaign, UrbanaIL, USA; ^3^Global Change and Photosynthesis Research Unit, United States Department of Agriculture, UrbanaIL, USA; ^4^Chinese Academy of Sciences–German Max Planck Society Partner Institute for Computational Biology, Shanghai Institutes for Biological Sciences, Chinese Academy of SciencesShanghai, China

**Keywords:** photosynthesis, solar energy conversion efficiency, chlorophyll, canopy light distribution, soybean

## Abstract

Reducing chlorophyll (chl) content may improve the conversion efficiency of absorbed photosynthetically active radiation into biomass and therefore yield in dense monoculture crops by improving light penetration and distribution within the canopy. The effects of reduced chl on leaf and canopy photosynthesis and photosynthetic efficiency were studied in two reportedly robust reduced-chl soybean mutants, *Y11y11* and *y9y9*, in comparison to the wild-type (WT) “Clark” cultivar. Both mutants were characterized during the 2012 growing season whereas only the *Y11y11* mutant was characterized during the 2013 growing season. Chl deficiency led to greater rates of leaf-level photosynthesis per absorbed photon early in the growing season when mutant chl content was ∼35% of the WT, but there was no effect on photosynthesis later in the season when mutant leaf chl approached 50% of the WT. Transient benefits of reduced chl at the leaf level did not translate to improvements in canopy-level processes. Reduced pigmentation in these mutants was linked to lower water use efficiency, which may have dampened any photosynthetic benefits of reduced chl, especially since both growing seasons experienced significant drought conditions. These results, while not confirming our hypothesis or an earlier published study in which the *Y11y11* mutant significantly outyielded the WT, do demonstrate that soybean significantly overinvests in chl. Despite a >50% chl reduction, there was little negative impact on biomass accumulation or yield, and the small negative effects present were likely due to pleiotropic effects of the mutation. This outcome points to an opportunity to reinvest nitrogen and energy resources that would otherwise be used in pigment-proteins into increasing biochemical photosynthetic capacity, thereby improving canopy photosynthesis and biomass production.

## Introduction

Increasing the yield potential (*Y*_p_) of important agronomic crops is imperative for meeting predicted future production needs. *Y*_p_ is the maximum possible regional yield for a given crop in the absence of biotic and abiotic stresses (Evans and Fischer, 1999), but as radiation, water, nutrients, etc., become limiting or there is pest/pathogen pressure, realized yields decrease, resulting in a yield gap (Lobell et al., 2009). *Y*_p_ for a given crop during a growing season is the product of several components: the incident solar radiation across the growing season (*S*_t_), the proportion of *S*_t_ that is photosynthetically active radiation (PAR; estimated as 0.487), the radiation interception efficiency (𝜀_i_), the conversion efficiency of intercepted radiation into biomass (𝜀_c_), and the partition efficiency of biomass into harvestable product (𝜀_p_; modified from Monteith, 1977). *S*_t_ and the proportion that is PAR vary but are largely predetermined by growing season length and location, although there can be substantial interannual variability in *S*_t_ at a given location (Monteith, 1965, 1972). Of the three efficiencies, plant breeders have already pushed 𝜀_i_ and 𝜀_p_ near their theoretical upper limits in highly productive crops in the best years (Evans, 1993; Hay, 1995; Sinclair, 1998). Breeding in soybean (*Glycine max* Merr.) has achieved 𝜀_i_ season averages of approximately 0.60–0.75 (Koester et al., 2014) with peak midseason 𝜀_i_ of >0.90 in modern cultivars (Dermody et al., 2008; Koester et al., 2014). 𝜀_p_ has reached values of 0.60 for soybean (Dermody et al., 2008; Koester et al., 2014), which is at or near the estimated theoretical maximum of ∼0.55–0.67 for major food crops (Austin et al., 1980; Bugbee and Monje, 1992; Khush, 1995; Smil, 1999; Hay and Porter, 2006; Prasad et al., 2006). These advancements in 𝜀_i_ and 𝜀_p_ leave only modest potential for further improvement of *Y*_p_ (Zhu et al., 2010). However, 𝜀_c_ operates substantially below the theoretical maxima for *C*_3_ (0.094) and *C*_4_ (0.123; Zhu et al., 2010) crop canopies and therefore limits yield potential (Zhu et al., 2008, 2010), especially in soybean where the maximum realized 𝜀_c_ (0.028) is estimated at less than a third of the *C*_3_ theoretical potential (Slattery and Ort, 2015).

Reducing leaf chlorophyll (chl) content has been proposed as a strategy to improve 𝜀_c_ in crop canopies. At low light levels, leaf photosynthesis (*A*_leaf_) in crops such as soybean responds linearly to light intensity, but at approximately 25% of full sunlight, or 500 μmol m^-2^ s^-1^ photosynthetic photon flux density (PPFD), the linear relationship between absorbed quanta and *A*_leaf_ begins to plateau (Long et al., 2006). Light in excess of photosynthetic capacity is then wasted through heat dissipation, or photoprotection, which reduces light use efficiency (Niyogi, 1999; Ort, 2001). At normal chl levels, individual soybean leaves absorb approximately 85–90% of incident PPFD, which results in the uppermost ∼25% of the canopy absorbing ∼75% of incoming light (Campbell and Norman, 1998), much of which is wasted due to light saturation of photosynthesis in these leaves. Meanwhile, at full sunlight, leaves below the uppermost 25% receive half or less of the light needed to saturate *A*_leaf_ and are therefore light limited (Long et al., 2006). By distributing light more proportionately throughout leaf layers (Zhu et al., 2010; Ort et al., 2011, 2015), absorbed PPFD could be used more efficiently by mitigating both light oversaturation at the top of canopy and light limitation within the canopy. It is likely that some crops overinvest in chl content to the detriment of light distribution in the canopy similar to the manner in which soybean overinvest in leaf area (Srinivasan et al., 2016). Therefore, decreasing leaf absorbance (leaf_abs_) through reduced chl content seems a potential strategy to achieve deeper light penetration into a crop canopy; thus, sun leaves would absorb only enough photons at mid-day to sustain maximum *A*_leaf_ while allowing more light to reach the lower canopy and stimulate *A*_leaf_ in shade leaves, thereby potentially improving canopy photosynthesis (*A*_can_), 𝜀_c_, and *Y*_p_. Experimental evidence supporting the principle of this notion has been found in similar or greater rates of *A*_leaf_ in various crops with substantial reductions in chl compared to their dark-green counterparts (Highkin et al., 1969; Benedict et al., 1972; Edwards et al., 1993; Habash et al., 1994; Li et al., 2013; Kirst et al., 2017). In soybean, greater rates of *A*_leaf_ on an absorbed photon basis were evident in light-green soybean leaves, and the increase in *A*_leaf_ correlated with a more even light distribution among chloroplasts within leaves (Slattery et al., 2016). An analogous alteration of light distribution could therefore occur among leaves within a canopy. In addition, dense mass cultures of truncated light antennae (*tla*) green algae mutants demonstrated increased light penetration and improved solar energy conversion efficiency (Melis, 1999; Polle et al., 2002; Mitra and Melis, 2008), which ultimately led to increased hydrogen production (Kosourov et al., 2011).

Decreasing chl content could also have other benefits at the canopy level. Reducing light absorption and thereby increasing albedo at the top of the canopy could decrease leaf temperature (*T*_leaf_) in the upper canopy, similar to the manner in which paraheliotropism reduces *T*_leaf_ in other species (Gamon and Pearcy, 1989). During times of above optimal temperatures, this should increase *A*_leaf_ by mitigating negative heat stress effects (Ainsworth and Ort, 2010) and in turn also improve water use efficiency (WUE). Cooler soybean canopies lower vapor pressure deficit, resulting in higher WUE (Baldocchi et al., 1985), which was reported for alfalfa with reduced chl content compared to the full green control (Estill et al., 1991). Greater light availability with depth in the canopy could also increase WUE by facilitating greater *A*_leaf_ in deeper layers where humidity is higher and therefore vapor pressure deficit is lower (Drewry et al., 2014; Ort and Long, 2014). If monoculture crops are overinvesting in chl biosynthesis, reallocation of nitrogen from an excess of pigment-protein complexes to other nitrogen-limited photosynthesis-related molecules might also be a benefit of chl reduction. In a modeling study, reallocating nitrogen resources among Calvin cycle enzymes predicted increased potential *A*_leaf_ without any additional nitrogen (Zhu et al., 2007). If nitrogen that would otherwise be used in pigment and pigment-proteins were reinvested in increased photosynthetic capacity, a similar increased nitrogen use efficiency would be expected.

Soybean is the world’s third most economically important commodity crop (FAO, 2012). At agricultural planting densities, soybean develops a dense canopy with a leaf area index (LAI) often greater than six. This creates a situation in which the majority of leaves are experiencing light levels below the light compensation point during most daylight hours and makes it an ideal candidate crop for testing the effects of reduced chl content on 𝜀_c_. A large number of chl-deficient mutants have been identified in soybean, and two chl-deficient soybean mutants with robust canopy growth, *Y11y11* and *y9y9*, were previously reported to have greater *A*_can_ compared to the nearly isogenic “Clark” wild-type (WT) throughout the growing season (Pettigrew et al., 1989). These mutants display a disproportionately large truncation in the antennae associated with photosystem II (PSII) compared to photosystem I (PSI; Ghirardi and Melis, 1988). This leads to higher chl *a*/*b* and PSII/PSI ratios, the latter of which serves to balance light absorption between the two photosystems (Eskins et al., 1983; Ghirardi and Melis, 1988). However, comprehensive studies of reduced chl effects on soybean at both the leaf and canopy scale have not yet been conducted in the same experiment. Therefore, the same light-green soybean mutants and the WT control were grown in the field during the 2012 growing season. The following year only the *Y11y11* mutant was grown with the WT so that a row spacing treatment could be added. These field experiments were used to characterize the light-green mutants and to investigate the impact that reducing chl content has on leaf and canopy photosynthesis, photosynthetic efficiency, and yield.

## Materials and Methods

### Site Description

Field experiments were conducted at the SoyFACE global change research facility at the University of Illinois at Urbana-Champaign (40°02′N, 88°14′W, 228 m above sea level) during the 2012 and 2013 growing seasons. The soil at this site is a deep and fertile Flanagan (fine, montmorillonitic, mesic aquic Argiudoll) with some low-lying blocks of Drummer [typic Haplaquoll; Rogers et al., 2004]. The site maintained a yearly maize-soybean rotation, and no nitrogen fertilizer was added prior to soybean planting in accordance with standard regional practices.

**FIGURE 1:**
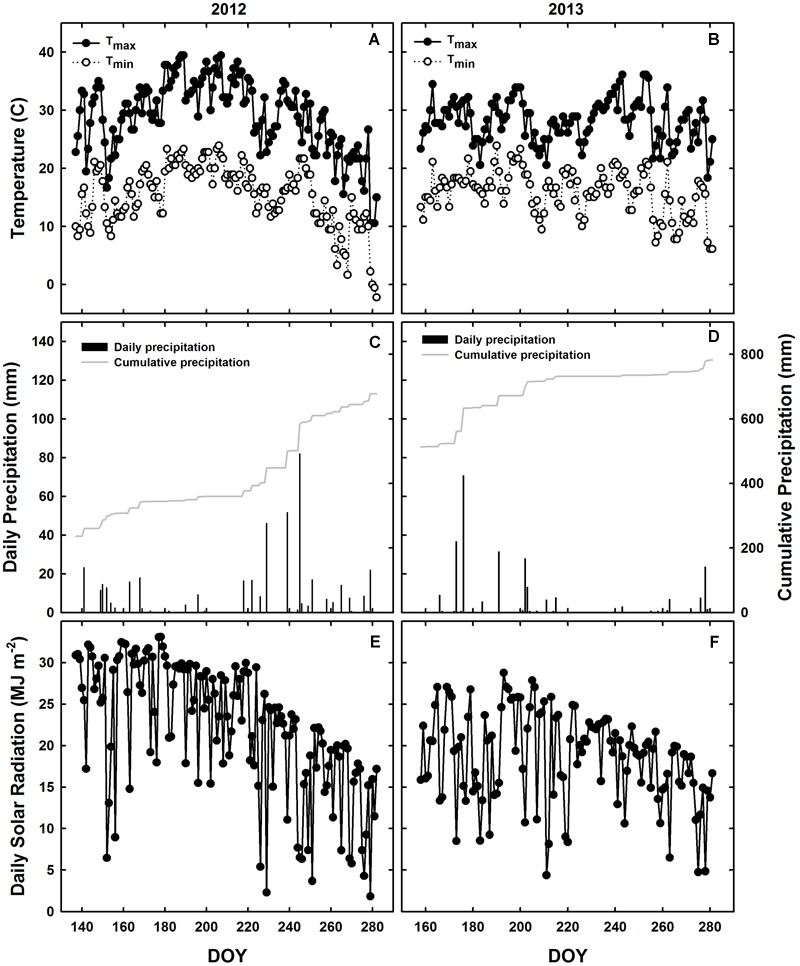
**Meteorological conditions during the 2012 and 2013 growing seasons in Champaign, IL, USA.** Daily observations from 2012 **(A,C,E)** and 2013 **(B,D,F)** are indicated for maximum (black circles) and minimum (white circles) temperatures **(A,B)**, daily precipitation during the growing season (black bars) and cumulative annual precipitation (gray line; **C,D**), and incident solar radiation **(E,F)**.

The experimental design consisted of a randomized complete block design with three replicates. WT soybean cultivar “Clark” and two nearly isogenic chl-deficient mutants, *Y11y11* and *y9y9* (Eskins et al., 1981), were grown in 2012. Only WT and *Y11y11* were grown in 2013 in order to accommodate a row spacing treatment. Plots in 2012 consisted of 16–2.74 m rows running north-south with a row spacing of 0.38 m. Planting density was 30 plants m^-2^. In 2013, a row spacing treatment was introduced by replicating the same design but adding a narrower row spacing treatment (0.19 m between rows) while maintaining a plant density of 30 plants m^-2^ (Supporting Information Figure S1). Since the *Y11y11* genotype segregates (1 dark green: 2 light green: 1 yellow plant), it was planted at a higher density to account for the removal of *Y11Y11* dark green and the seedling lethal *y11y11* yellow plants before determining the final plant density. Planting in 2012 occurred on 16 May [day of year (DOY) 137], and harvest occurred on 17 October (DOY 291). In 2013, seeds were sown in the 0.38 m row spacing treatment on 7 June (DOY 158) with a cone planter. The narrow row spacing treatment was planted 1 day later on 8 June (DOY 159) with a push-planter. All plants were harvested on 11 October (DOY 284). Daily meteorological data spanning the growing season (planting to harvest) were obtained from the Illinois Climate Network monitoring station ∼1.5 km from the field site (**Table 1** and **Figure 1**; Angel, 2009).

**Table 1:** Meteorological conditions during the 2012 and 2013 soybean growing seasons (planting to harvest) in Champaign, IL, USA.

Year	Row space (m)	Planting date	Emergence date	Harvest date	Precipitation^a^ (mm)	*T*_max_^a^(°C)	*T*_min_^a^(°C)	*T*_mean_^a^ (°C)	Solar radiation^a^ (MJ m^-2^)
2012	0.38	16 May	25 May	17 Oct	517^†^	28.4	14.9	21.4	3,337
2013	0.38	7 Jun	17 Jun	16 Oct	271	28.2	15.7	21.6	2,396
2013	0.19	8 Jun	21 Jun	16 Oct	271	28.3	15.7	21.6	2,380

*Total precipitation, average maximum (*T*_*max*_), minimum (*T*_*min*_), and mean temperature (*T*_*mean*_), and total available solar radiation over each growing season (planting to harvest) are indicated along with planting, emergence, and harvest dates for each experiment. ^a^Data from the Illinois Climate Network (http://www.isws.illinois.edu/warm/datatype.asp) were used to find mean temperatures and sums of precipitation and solar radiation from each growing season. ^†^Precipitation (481 mm) + two irrigations of 18 mm each during mid-July.*

### Gas Exchange Measurements

#### Diurnal Leaf Gas Exchange

All leaf gas exchange measurements were conducted with open path gas exchange systems equipped with leaf chamber fluorometers (LI-6400, LI-COR, Lincoln, NE, USA). Diurnal gas exchange measurements (5–6 time points at 2 h intervals throughout the day) were conducted six times during the 2012 growing season and five times during 2013 on sun leaves. A diurnal was conducted on shade leaves after canopy closure in 2012 as well. Sun leaves were designated as the youngest, fully expanded leaves exposed to full sunlight throughout the day. Shade leaves were designated as 3–4 nodes below the sun leaf on the same plant. Measurement chamber conditions were set to ambient PPFD and 60–70% relative humidity. For shade leaves, PPFD was measured within the canopy using a 1 m long quantum sensor (LI-190, LI-COR, Lincoln, NE, USA) before each set of measurements. The sensor was inserted into the canopy at the height of the shade leaves and at multiple locations within each block. The average light level across all blocks within each genotype was then used for that time point. Block temperature of the gas exchange system was set to ambient air temperature, and reference CO_2_ concentration ([CO_2_]) was set to 400 ppm. The daily integral of *A*_leaf_ (*A*′) was determined as in Rogers et al. (2004) for each replicate, after which statistical analyses were conducted (see below). Daily means of *A*_leaf_, *g*_s_, intrinsic water use efficiency (iWUE; calculated as *A*_leaf_/*g*_s_ for each point measurement before statistically analyzing as described below), and *T*_leaf_ (measured by the LI-6400 LI-COR, Lincoln, NE, USA) were also determined for sun leaves.

#### Light Response of Leaf Photosynthesis

Photosynthetic light response (*A/Q*) curves were conducted on sun leaves in the field at midday during the V5 (five true leaves) developmental stage in 2012 and during the V5, R1/R2 (flowering), and R5 (pod filling) developmental stages (Fehr et al., 1971) in 2013. Shade leaf *A/Q* measurements were only conducted in 2013 after canopy closure (R1/R2 and R5). Sun and shade leaves were designated as described above. Curves consisted of 12 points spanning from 0 to 2000 μmol m^-2^ s^-1^ PPFD. Dark-adapted minimal fluorescence (*F*_o_) and maximal fluorescence (*F*_m_) were measured pre-dawn the same day as *A/Q* measurements were conducted. Light-adapted steady state fluorescence (*F*_s_′), minimal fluorescence (*F*_o_′), and maximal fluorescence (*F*_m_′) were measured on each leaf during *A*/*Q* measurements according to Baker (2008). The operating efficiency of photosystem II (ϕ*PSII*) was calculated as (*F*_m_′*-F*_s_)*/F*_m_′ and non-photochemical quenching (*NPQ*) was calculated as (*F*_m_*-F*_m_′)/*F*_m_′. The electron transport rate through PSII (*ETR*) was calculated as PPFD^∗^Leaf_abs_^∗^*f*_PSII_^∗^ϕ*PSII* where *f*_PSII_ is the fraction of absorbed PPFD that goes to PSII (Baker, 2008). Maximum rate of photosynthesis (*A*_sat_) was determined by fitting a non-rectangular curve to the data (SigmaPlot, Systat Software Inc., San Jose, CA, USA). Maximum quantum efficiency (ϕ*CO*_2_) was determined as the slope of the linear fit of *A*_leaf_ versus absorbed PPFD (Proc Reg; SAS 9.4, SAS Institute, Cary, NC, USA) at low light using data from light levels at or above the light compensation point to avoid any potential effects on the slope due to the Kok effect (Kok, 1948). Proc Loess (SAS 9.4; SAS Institute, Cary, NC, USA) was used to determine 90% confidence intervals for all *A*/*Q* data where non-overlapping intervals indicated significant differences. The relationship between *A*_sat_ and chl content across all genotypes and growing seasons was also plotted with a second order logarithmic function (SigmaPlot, Systat Software Inc., San Jose, CA, USA).

#### CO_2_ Response of Leaf Photosynthesis

Photosynthetic [CO_2_] response (*A/C*_i_) curves were conducted every 2 weeks throughout the 2012 growing season and during the V5, R1/R2, and R5 developmental stages in 2013. Measurements were conducted on sun leaves, and shade leaves were also measured in 2013 after canopy closure (R1/R2 and R5 stages). Maximum carboxylation rates of Rubisco (*V*_c,max_), maximum electron transport rates (*J*_max_), and the intercellular [CO_2_] at the inflection point between Rubisco and RuBP limited *A*_leaf_ (*C*_i,inflection_) were determined according to Long and Bernacchi (2003). *V*_c,max_ versus chl content and *J*_max_ versus chl content were also plotted in the same manner as the *A*_sat_ versus chl content relationship described above.

#### Leaf Dark Respiration

Dark respiration (*R*_d_) was measured 1–3 h after dusk using a LI-6400 equipped with a specially designed leaf chamber able to enclose an entire trifoliate leaf (Gillespie et al., 2012). Measurements were conducted at the three developmental stages in 2013 on sun and shade leaves as described above. After measurements, leaves were detached and leaf area was measured using a leaf area meter (LI-3100, LI-COR, Lincoln, NE, USA) in order to calculate *R*_d_ on a leaf area basis.

#### Midday Canopy Photosynthesis

*A*_can_ was measured at midday using a portable chamber on DOY 197 (V5), 213 (R1/R2), 221 [R3/R4 (pod initiation)], and 242 (R5) during 2013 on wide row widths planting in a manner similar to Prater et al. (2006). A chamber (0.914 m × 1.02 m base × 1.37 m height) with clear plastic siding was equipped with mixing fans and a rubber gasket on the bottom edge. An open path infrared gas analyzer (LI-7500, LI-COR, Lincoln, NE, USA) was mounted just above the canopy height within the chamber and was connected to a data logger (LI-7550, LI-COR, Lincoln, NE, USA) outside of the chamber. To reduce soil disturbance and prevent leakage during measurements, aluminum frames with vertical sides and a flat surface on top matching the dimensions of the chamber base (0.914 m × 1.02 m) were inserted into the soil in the area of measurement at least 1 day before measurements. The bottom surface of the chamber was lowered onto the flat top surface of the frame, rather than the uneven soil surface, with a seal created by the rubber gasket. CO_2_ drawdown was measured on the two rows of plants encompassed within the chamber within 1 min of lowering the chamber over the canopy and onto the frames to minimize any microclimate effects. Soil respiration was measured using an infrared gas analyzer equipped with a soil CO_2_ flux chamber (LI-6400-09, LI-COR, Lincoln, NE, USA). Soil respiration measurements were conducted in two locations within the measured *A*_can_ area within 1 h prior to or immediately following the chamber measurements to account for any changes in [CO_2_] within the chamber due to soil CO_2_ flux. One row of plants from within the chamber area was used for biomass harvests (see below), and total leaf area within the chamber was estimated from those measurements. Canopy CO_2_ assimilation rates were calculated after accounting for soil respiration rates and adjusted to a leaf area basis.

### Leaf Tissue Sampling and Biomass Harvests

#### Leaf Tissue Sampling

Leaf disks 2 cm in diameter were collected at midday during each diurnal and dried to determine specific leaf weight (SLW; g m^-2^). Leaf disks 1 cm in diameter were collected at midday during each diurnal to determine chl content, chl *a/b* ratios, and total carotenoid content using the methods of Lichtenthaler (1987) and Porra et al. (1989). Near the end of the 2013 season during developmental stage R5, 2 cm leaf disks were taken from leaves at the top (uppermost 0.25m), middle (0.25–0.50 m from the top of the canopy), and bottom (0.50–0.75 m from the top of the canopy) of the canopy to determine integrated WUE using isotope analyses (Farquhar and Richards, 1984). The samples were dried and ground to a powder, after which an elemental analyzer (Elemental Combustion System 4010, Costech Analytical Technologies, Inc., Valencia, CA, USA) in parallel with an isotope ratio mass spectrometry system (Finnigan Delta V Advantage Mass Spectrometer, Thermo Fisher Scientific, Waltham, MA, USA) were used to determine δ^13^C on a per mass basis.

#### Leaf Absorbance Measurements

Leaf_abs_ was measured during the three developmental stages of 2013 at various heights within the canopy using an integrating sphere (Spectroclip-JAZ-TR, Ocean Optics, Duiven, The Netherlands). Leaf_abs_, or the fraction of light absorbed, was calculated as

$${\text{Leaf}}_{\text{abs}}=I_{\text{o}}-I_{\text{t}}-I_{\text{r}}$$

where *I*_o_ is incident radiation, *I*_t_ is transmitted radiation, and *I*_r_ is reflected radiation. Absorbance of the blue (460 nm) and red (635 nm) wavelengths emitted from LEDs within the open gas exchange chambers (LI-6400, LI-COR, Lincoln, NE, USA) was used to calculate total absorbed PPFD during *A/Q* measurements. Since leaf_abs_ was not measured in 2012, the relationships between chl content and blue and red light absorbance from 2013 (Supporting Information Figure S2) were used to estimate absorbed PPFD during 2012 *A/Q* measurements.

#### Biomass Determination

Aboveground biomass harvests were conducted every 10–14 days each season by harvesting 1 m of a row in each plot at soil height while avoiding plot borders or previous harvest locations. Plant height was measured on three of the plants, and the number of plants per meter of row was recorded. Leaf area per plant was determined for five plants in each plot using a leaf area meter (LI-3100, LI-COR, Lincoln, NE, USA) and adjusted for total plant number to determine total leaf area within the canopy chamber (see above). Stems, leaves, and pods were then separated and dried at 65–70°C for 3 days to determine dry weights. Stem and leaf dry weights were converted to MJ of energy per land area using the tissue-specific energy contents from Amthor et al. (1994). Pod energy at various reproductive stages was determined in 2013 using a bomb calorimeter with a benzoic acid standard (Model 1261, Parr Instrument, Moline, IL, USA). This was used to convert pod mass to pod energy on a land area basis for each reproductive stage after pod initiation in both 2012 and 2013.

### 𝜀_i_, 𝜀_c_, 𝜀_p_, and Yield

Daily canopy light interception fraction and season-long interception efficiency, 𝜀_i_, were determined as the fraction of available PAR that was absorbed (APAR) by the canopy. APAR was calculated as

$$\text{APAR}=I_{\text{o}}-(I_{\text{t}}+I_{\text{r}})$$

where *I*_o_ was incident PAR measured above the canopy with an upright quantum sensor, *I*_t_ was transmitted PAR measured at soil level using a line sensor, and *I*_r_ was reflected PAR measured with an inverted quantum sensor above the canopy. All data were collected using line (model SQ-311) and quantum (model SQ-110) sensors (Apogee Instruments, Logan, UT, USA) that had been calibrated with a high precision quantum sensor (LI-190, LI-COR, Lincoln, NE, USA) at the beginning of the season. All data were logged every 10 s using a datalogger (model CR3000 in 2012 and model CR10X in 2013, Campbell Scientific, Logan, UT, USA). Measurements began on DOY 180 in 2012 and DOY 189 in 2013 and corresponded to the V5 developmental stage. The energy conversion efficiency (𝜀_c_) was determined as the slope of accumulated aboveground biomass energy regressed on accumulated APAR from early vegetative stages to peak biomass energy. 𝜀_p_ was determined as the ratio of seed energy: total aboveground plant energy at harvest maturity. Yield and seed mass were determined after harvesting and threshing seeds from pods of four complete rows per plot in each experiment.

### Statistical Analyses

Statistical analyses were conducted on the plot means using a mixed model ANOVA (Proc Mixed, SAS 9.4; SAS Institute, Cary, NC, USA) with genotype, time of day, and DOY considered fixed effects and block and block by genotype effects considered random. SLW, chl content, chl *a/b, A*′, mean daily *A*_leaf_, mean daily *g*_s_, mean daily iWUE, mean daily *T*_leaf_, leaf_abs_, *V*_c,max_, *J*_max_, *C*_i,inflection_, and LAI were analyzed as repeated measures with DOY as the repeated factor. Least squared means are reported and shown in figures with the associated standard errors. 𝜀_c_ regressions and comparisons were performed on pooled plot data points (Proc Reg, SAS 9.4; SAS Institute, Cary, NC, USA). To reduce the probability of type II errors, an α of 0.1 was used to determine significance.

## Results

### Weather Conditions Differed Greatly between 2012 and 2013 but Still Resulted in Drought Conditions in Both Seasons

Planting occurred 3 weeks later in 2013 as compared to 2012 (**Table 1**), but mean daily temperatures were similar across both growing seasons (**Table 1** and **Figures 1A,B**). Overall precipitation during 2012 was almost double that of 2013 (**Table 1**), but annual cumulative precipitation was less than half as much at the start of the 2012 growing season as compared to the 2013 season (**Figures 1C,D**). Low precipitation amounts prior to planting in 2012 and the fact that most of the 2012 precipitation fell late in the season led to a significant drought from early to mid-season in 2012 (**Figure 1C**), which was prior to developmental stage R5 (pod filling; data not shown). A moderate drought also occurred late in the 2013 growing season (**Figure 1D**), the beginning of which corresponded to developmental stage R3 (pod initiation; data not shown). Total *S*_t_ (from planting to harvest) was almost 30% lower during 2013 as compared to 2012 (**Figures 1E,F** and **Table 1**) and was slightly lower in the narrow row spacing due to planting 1 day after the wide row spacing (**Table 1**). Lower *S*_t_ in 2013 was only partially attributed to a later planting date in 2013 compared to 2012 (**Table 1**). Total monthly *S*_t_ was also greater during the main growing months of 2012. Total monthly *S*_t_ was 36% greater in June, 28% greater in July, and 8% greater in August of 2012 compared to the corresponding months in 2013.

### SLW and Pigment Concentrations Were Significantly Altered in the Mutants Compared to WT, but Reductions in Leaf_abs_ Were Less Pronounced

**FIGURE 2:**
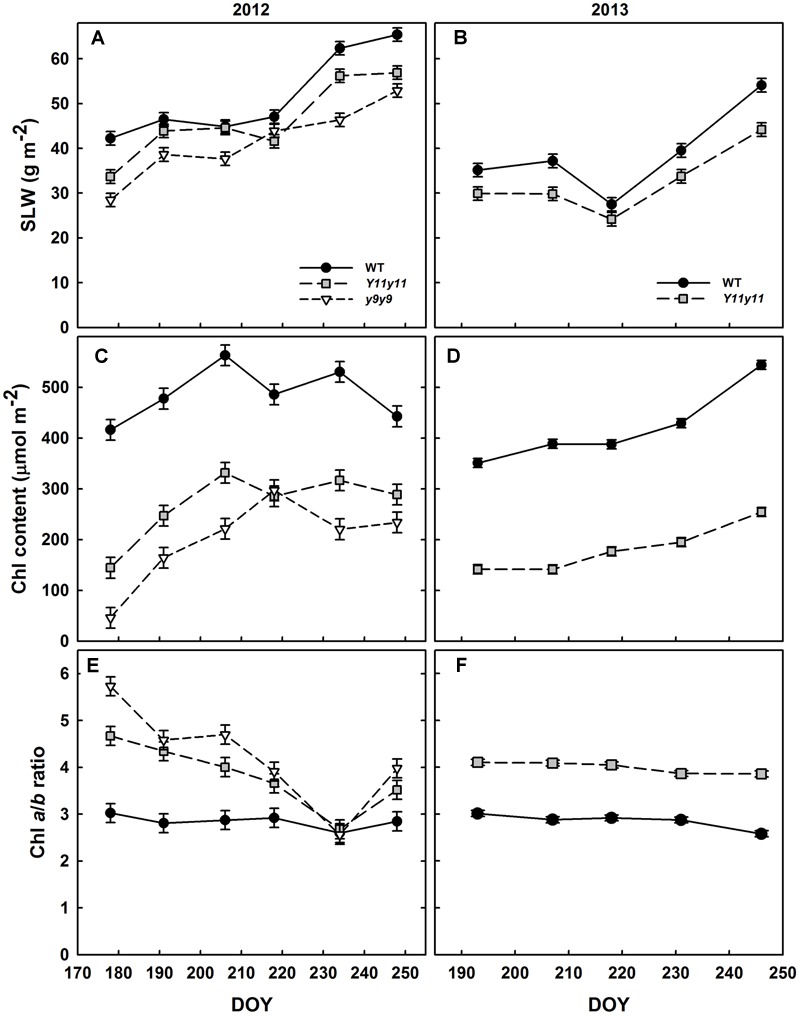
**Soybean specific leaf weight (SLW) and chlorophyll (chl) from two growing seasons.** Mean SLW **(A,B)**, total leaf chl **(C,D)**, and chl *a*/*b* ratios **(E,F)** are indicated across the 2012 **(A,C,E)** and 2013 **(B,D,F)** growing seasons for WT dark-green soybean (black circles) and two chl-deficient soybean mutants (*Y11y11* = gray squares; *y9y9* = white triangles). Error bars represent standard errors (*n* = 3).

Specific leaf weight was significantly reduced in the chl mutants in both growing seasons (Supporting Information Table S1). *y9y9* SLW was significantly lower than WT SLW on all days but one (DOY 218) during the 2012 season, whereas *Y11y11* SLW was significantly reduced on all days but DOY 206 and 218 (**Figure 2A**). In 2013, *Y11y11* SLW was significantly reduced compared to WT on all days except DOY 218 (**Figure 2B**).

The mutations in *Y11y11* and *y9y9* caused clear reductions in chl content while increasing the chl *a*/*b* ratios and decreasing total carotenoids. Chl content was significantly affected by the genotype by DOY interaction in both years (Supporting Information Table S1). Mean chl content was significantly reduced by approximately 45–60% in *Y11y11* and *y9y9* across 2012 (**Table 2** and **Figure 2C**). Throughout 2013, *Y11y11* chl content was reduced on average by 57% (**Table 2** and **Figure 2D**). However, mutant chl content was lowest early in both seasons and increased with development (**Figures 2C,D**). Chl *a*/*b* ratios were significantly affected by genotype by DOY interactions in 2012, but only main effects were significant in 2013 (Supporting Information Table S1). In both seasons the mutant chl *a*/*b* ratios were generally greater than WT ratios (**Table 2** and **Figures 2E,F**), but the differences decreased as the 2012 season progressed (**Figure 2E**), whereas the chl *a*/*b* ratios were consistent in both *Y11y11* and WT throughout the 2013 growing season (**Figure 2F**). Total carotenoid content was significantly reduced by >30% in *Y11y11* and >40% in *y9y9* compared to WT (**Table 2** and Supporting Information Table S1).

**Table 2:** Light- and dark-green soybean leaf pigment and physiological parameters from two growing seasons and two leaf levels.

			2012				2013		
Leaf	Parameter		WT	*Y11y11*	*y9y9*	MSE	WT	*Y11y11*	MSE
Sun	SLW	(g m^-2^)	51.4	46.1^∗^	41.3^∗^	0.78	38.7	32.4^∗^	0.61
	Chl content	(μmol m^-2^)	486	269^∗^	197^∗^	13	420	182^∗^	4.9
	Chl *a*/*b*	–	2.84	3.81^∗^	4.24^∗^	0.065	2.85	3.99^∗^	0.036
	Carotenoids	(g m^-2^)	59.3	40.1^∗^	35.3^∗^	2.5	55.3	35.6^∗^	1.0
	Leaf_abs_	–	–	–	–	–	0.86	0.72^∗^	0.58
	*A*′	(mol m^-2^ d^-1^)	0.787	0.854^∗^	0.758^∗^	0.011	0.834	0.832	0.006
	*A*_leaf_	(μmol m^-2^ s^-1^)	18.1	19.6^∗^	17.5	0.26	20.3	20.3	0.15
	*g*_s_	(mol H_2_O m^-2^ s^-1^)	0.225	0.268^∗^	0.300^∗^	0.014	0.641	0.796^∗^	0.011
	iWUE	(μmol mol^-1^)	95.9	87.5	73.0^∗^	3.5	35.0	27.5^∗^	0.34
	*T*_leaf_	(°C)	29.8	29.4	29.0	0.081	23.9	23.4	0.057
	*V*_c,max_	(μmol m^-2^ s^-1^)	121	127	116	3.3	107	106	3.5
	*J*_max_	(μmol m^-2^ s^-1^)	162	167	158	2.8	166	178^∗^	3.3
	*C*_i,inflection_	(μmol mol^-1^)	187	176	182	6.5	182	233^∗^	15
	*A*_sat_	(μmol m^-2^ s^-1^)	33.2	47.4^∗^	28.6	4.3	34.5	32.6	0.52
	ϕ*CO*_2_	–	0.068	0.061	0.036^∗^	0.00004	0.054	0.061	0.005
	*R*_d_	(μmol m^-2^ s^-1^)	–	–	–	–	-1.26	-1.10^∗^	0.027
Shade	Chl content	(μmol m^-2^)	558	242^∗^	105^∗^	20	395	168^∗^	21
	Chl *a*/*b*	–	2.27	3.26^∗^	4.46^∗^	0.15	2.67	3.37^∗^	0.16
	*A*′	(mol m^-2^ d^-1^)	0.174	0.194	0.185	0.016	–	–	–
	*V*_c,max_	(μmol m^-2^ s^-1^)	–	–	–		122	97.2	5.7
	*J*_max_	(μmol m^-2^ s^-1^)	–	–	–		188	164	15
	*C*_i,inflection_	(μmol mol^-1^)	–	–	–		206	247	27
	*A*_sat_	(μmol m^-2^ s^-1^)	–	–	–		30.9	27.2	3.5
	ϕ*CO*_2_	–	–	–	–		0.060	0.060	0.004
	*R*_d_	(μmol m^-2^ s^-1^)	–	–	–		-0.655	-0.542	0.060

*Growing season means are reported for variables related to physical leaf properties, diurnal measurements, photosynthetic response curves, and dark respiration rates.*

*Significant differences in relation to the WT are indicated with an asterisk at *p* < 0.1. Mean square error (MSE) is reported from each ANOVA. Dashes indicate effects not measured.*

**FIGURE 3:**
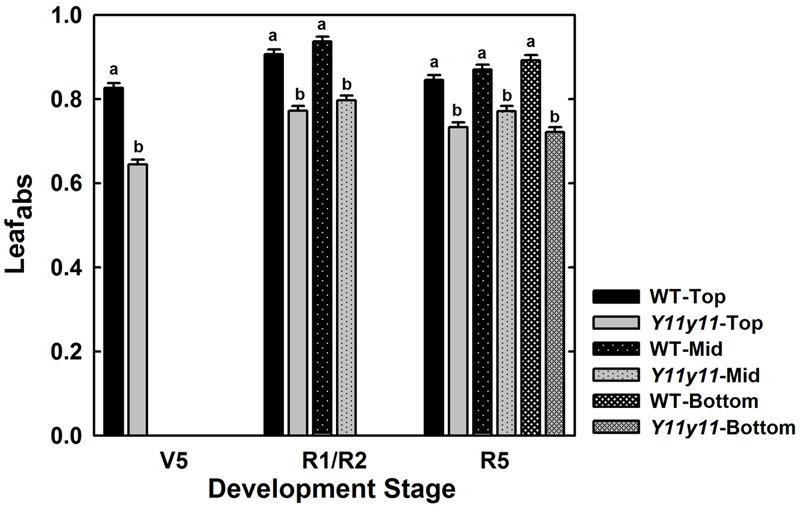
**Leaf absorbance as a function of canopy position across the 2013 season.** The fraction of absorbed photosynthetically active radiation (PAR) is indicated for WT (black) and *Y11y11* (gray) at the top (solid), middle (dotted), and bottom (grid) of the canopy throughout the 2013 season. Error bars represent the standard errors (*n* = 3). Letters indicate significant differences within developmental stage (α = 0.1) when present.

Although *Y11y11* chl content was reduced by approximately half in 2013, sun leaf_abs_ declined by only 16.6% compared to WT (**Figure 3** and **Table 2**). Leaf_abs_ was lowest for both WT and *Y11y11* during V5, at which time *Y11y11* leaf_abs_ was only 78% of the WT (**Figure 3**). Leaf_abs_ increased with development in both genotypes, but *Y11y11* leaf_abs_ was approximately 85% of the WT during reproductive stages (**Figure 3**). Leaf_abs_ also increased with depth in the canopy except for the lowest layer of the *Y11y11* canopy during R5 (**Figure 3**).

### Diurnal Measurements Indicated Transiently Greater *A*′ in Mutant Sun Leaves

**FIGURE 4:**
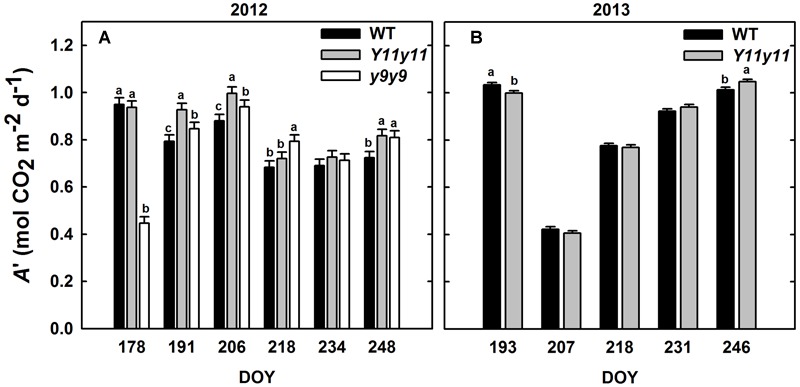
**Daily integrals of photosynthesis (*A*′) from two growing seasons.** Diurnal gas exchange measurements were used to calculate *A*′ in 2012 **(A)** and 2013 **(B)** for WT (black), *Y11y11* (gray), and *y9y9* (white) soybean. Error bars represent the standard errors (*n* = 3). Letters indicate significant differences within DOY (α = 0.1) when present.

Sun leaf *A*′ was significantly affected by genotype by DOY interactions in both years (Supporting Information Table S1). *Y11y11 A*′ was significantly greater than WT *A*′ on 3 days during the 2012 growing season (DOY 191, 206, 248; **Figure 4A**). *y9y9 A*′ was significantly lower than the control on the 1st day of measurements in 2012 but became significantly greater on DOY 191, 206, 218, and 248 (**Figure 4A**). When averaged over all measurement days from the 2012 growing season, *Y11y11 A*′ was 8.6% greater than WT *A*′, but *y9y9 A*′ was reduced by 3.6% (**Table 2**), mainly due to extremely low *A*′ on DOY 178 (**Figure 4A**). In 2013, *Y11y11 A*′ was significantly lower than the control on DOY 193 and significantly greater on DOY 246 (**Figure 4B**), and there was no significant difference between the mean season *A*′ (**Table 2**).

Shade leaf *A*′ was similar among all genotypes when measured in 2012 (**Table 2** and Supporting Information Table S1). Although *Y11y11 A*_leaf_ was significantly greater than the other two genotypes at the 1200 time point and *y9y9 A*_leaf_ was significantly greater than WT and *Y11y11* at the 1600 time point (Supporting Information Figure S3A), these increases were not large enough to significantly affect *A*′ (**Table 2**). Incident PPFD within the leaf chamber was based on light measurements within each canopy before each set of gas exchange measurements and was higher for the mutants as compared to the WT (Supporting Information Figure S3B). The reduced chl content of the *Y11y11* mutant, however, resulted in a similar amount of absorbed PPFD as compared to WT (Supporting Information Figure S3C).

### *A*_leaf_ and *g*_s_ were Greater in Mutant Sun Leaves whereas iWUE Was Reduced Despite Lower *T*_leaf_

**FIGURE 5:**
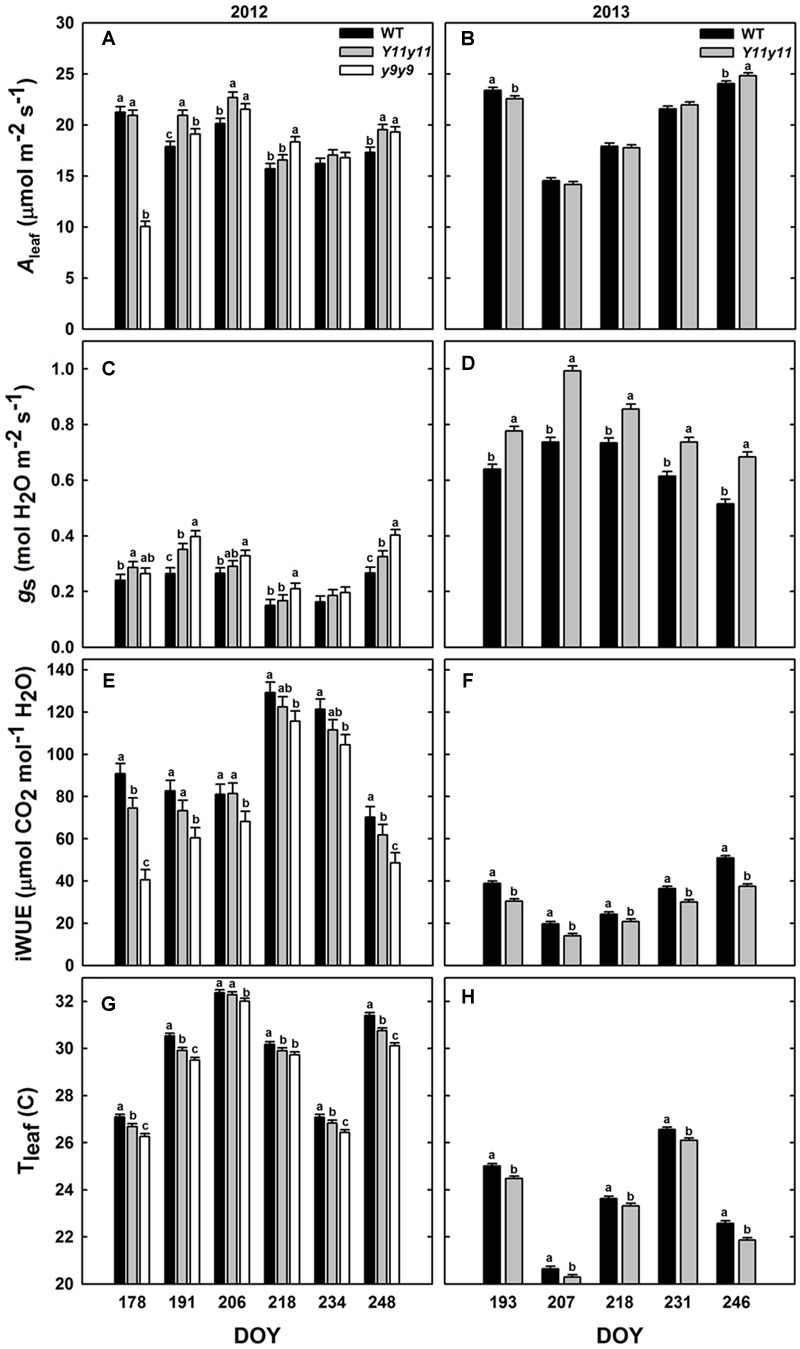
**Diurnal measurements in sun leaves from two growing seasons.** Bars represent daily means of photosynthesis (*A*_leaf_; **A,B**), stomatal conductance (*g*_s_; **C,D**), intrinsic water use efficiency (iWUE; **E,F**), and leaf temperature (*T*_leaf_; **G,H**) across the 2012 **(A,C,E,G)** and 2013 **(B,D,F,H)** growing seasons for WT dark green soybean (black) and two chl-deficient soybean mutants (*Y11y11* = gray; *y9y9* = white). Error bars represent the standard errors (*n* = 3). Letters indicate significant differences within DOY (α = 0.1) when present.

Diurnal measurements were used to calculate the daily means of *A*_leaf_, *g*_s_, iWUE, and *T*_leaf_ in sun leaves. Mean daily *A*_leaf_ was significantly affected by genotype by DOY interactions in 2012, but only DOY was significant in 2013 (Supporting Information Table S1). The within-day relationships between WT and mutant *A*_leaf_ were similar to those listed above for *A*′. In 2012, *y9y9 A*_leaf_ was significantly lower than WT only on DOY 178 but was significantly greater than WT on DOY 191, 206, 218, and 248 (**Figure 5A**). *A*_leaf_ in *Y11y11* was significantly greater than WT on DOY 191, 206, and 248 in 2012 (**Figure 5A**), leading to a significant 8.5% increase in mean season *A*_leaf_ in *Y11y11* (**Table 2**). In 2013, *Y11y11 A*_leaf_ was significantly lower than WT on DOY 193 but greater on DOY 246 (**Figure 5B**), resulting in no significant changes in season-long *A*_leaf_ (**Table 2**). *g*_s_ and iWUE were also significantly affected by the interaction effect (Supporting Information Table S1), but contrary to expectations, *g*_s_ was approximately 20–30% greater in the mutants across both seasons (**Table 2** and **Figures 5C,D**), resulting in lower mutant iWUE across both seasons (**Table 2** and **Figures 5E,F**). The diurnal iWUE data were supported by δ^13^C signature, in which the mean *Y11y11* signature across leaf position (-28.8‰) was significantly lower than the mean WT signature (-27.9‰; *p* < 0.0001; Supporting Information Figure S4). This indicated greater ^13^C discrimination in *Y11y11* and therefore higher *g*_s_ over the integral of leaf development across three layers of the canopy (Supporting Information Figure S4). *T*_leaf_ was significantly affected by the interaction between genotype and DOY in 2012 (*p* < 0.01) and the separate effects of genotype (*p* < 0.0001) and DOY (*p* < 0.0001) in 2013 (Supporting Information Table S1). As predicted, WT *T*_leaf_ was generally greater than mutant *T*_leaf_ in both seasons (**Table 2** and **Figures 5G,H**), which correlated with lower leaf_abs_ (**Figure 3**) but also greater transpiration (data not shown) in the mutants, which correlated with the higher *g*_s_.

### *Y11y11* Sun Leaves Demonstrated Greater Light Use Efficiency Compared to WT Early in the Growing Season

**FIGURE 6:**
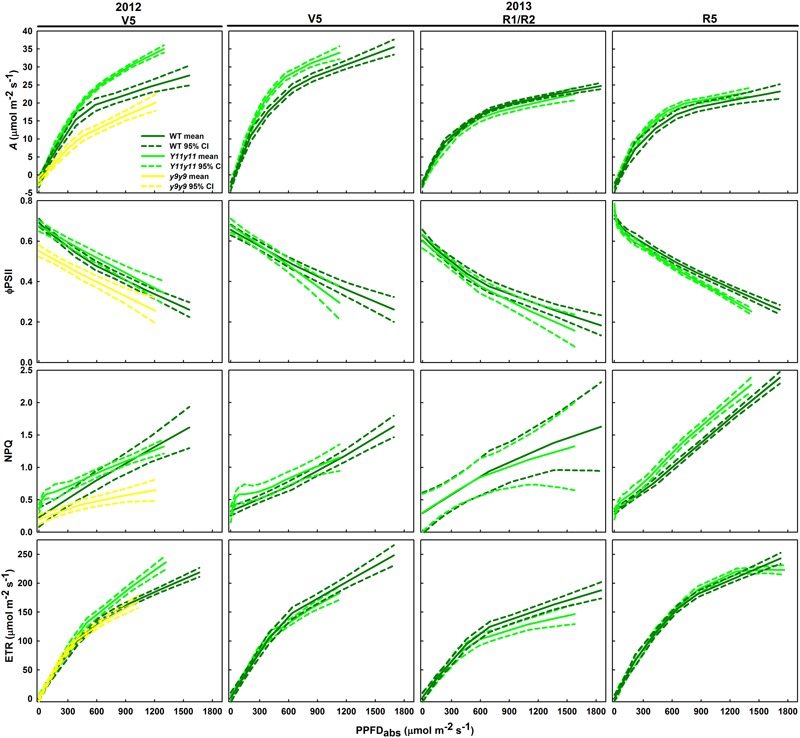
**Light response curves from various developmental stages across two growing seasons in sun leaves.** *A*_leaf_, ϕ*PSII, NPQ*, and *ETR* as a function of absorbed PPFD are shown in sun leaves during V5 of 2012 and during V5, R1/R2, and R5 of 2013. Means (solid line) and 90% confidence intervals (dashed lines) are indicated for each genotype (*n* = 3).

Since leaf_abs_ significantly differed across genotype and DOY, *A*/*Q* measurements were based on absorbed PPFD instead of incident PPFD. In both seasons, *Y11y11* sun leaves reached greater rates of *A*_leaf_ with fewer absorbed photons at high light levels during the V5 stage, demonstrating greater light use efficiency (**Figure 6**). Genotype main effects on sun leaf *A*_sat_ were significant in both years (Supporting Information Table S1). *Y11y11* sun leaves had a 43% higher *A*_sat_ early in 2012, whereas *y9y9 A*_sat_ was lower by 14% compared to WT (**Tables 2, 3**). In 2013, significant differences in *A*_sat_ occurred during R1/R2 (**Table 3**) but did not result in season-long differences (**Table 2**). The relationship between *A*_sat_ and chl shows a steep increase at chl contents less than 100 μmol m^-2^ and a more graduate decline with chl contents greater than 200 μmol m^-2^ (Supporting Information Figure S5A). ϕ*CO*_2_ was greater in *Y11y11* sun leaves compared to WT during the V5 stage of 2013, but season averages did not differ between the two genotypes in either year (**Tables 2, 3** and Supporting Information Table S1). On the other hand, *y9y9* sun leaf ϕ*CO*_2_ was significantly impaired early in 2012 when chl content was severely reduced (**Tables 2, 3**). Genotype effects were not significant within shade leaf *A*_sat_ or ϕ*CO*_2_ analyses in 2013 (**Tables 2, 3** and Supporting Information Table S1).

**Table 3:** Parameters from photosynthetic light response (*A*/*Q*) curves in two growing seasons.

		2012			2013			
		Sun			Sun		Shade	
Parameter	Stage	WT	*Y11y11*	*y9y9*	WT	*Y11y11*	WT	*Y11y11*
*A*_sat_	V5	33.2b	47.4a	28.6b	42.1a	41.0a	–	–
	R1/2	–	–	–	31.6a	28.1b	27.2a	19.1a
	R5	–	–	–	29.9a	27.6a	34.5a	35.4a
	MSE	4.3			1.3		4.9	
ϕ*CO*_2_	V5	0.068a	0.061a	0.036b	0.067b	0.081a	–	–
	R1/2	–	–	–	0.047a	0.043a	0.063a	0.056a
	R5	–	–	–	0.048a	0.058a	0.057a	0.064a
	MSE	0.00004			0.003		0.005	

*Parameters were calculated from photosynthesis versus absorbed PPFD. *A*_*sat*_ (μmol m^-*2*^ s^-*1*^) was calculated after fitting the data to a non-rectangular hyperbola. ϕ*CO*_2_ was the slope of the linear portion at low light. *A*/*Q* curves were only conducted in vegetative (V5) growth in 2012, whereas 2013 curves were conducted in V5 and two reproductive stages: flowering (R1/2) and pod-fill (R5). Mean square error (MSE) is reported from each ANOVA. Different letters represent significant differences at α = 0.1.*

### Chl Fluorescence Parameters Varied with Chl Content and Developmental Stage

Sun leaf ϕ*PSII* was significantly lower in *y9y9* in 2012 and *Y11y11* during R5 of 2013 (**Figure 6**). In 2012, the decrease in *y9y9* ϕ*PSII* was accompanied by substantially lower *NPQ* compared to the other two genotypes in mid to high light conditions (**Figure 6**). However, *NPQ* was similar between WT and *Y11y11* early in both seasons and greater in *Y11y11* during R5 in 2013 at mid to high light levels (**Figure 6**). *Y11y11 ETR* was greater than WT *ETR* at high light levels in 2012, but there were no significant differences in 2013 (**Figure 6**).

### Chl Reductions Had Little Effect on Biochemical Photosynthetic Capacity

**FIGURE 7:**
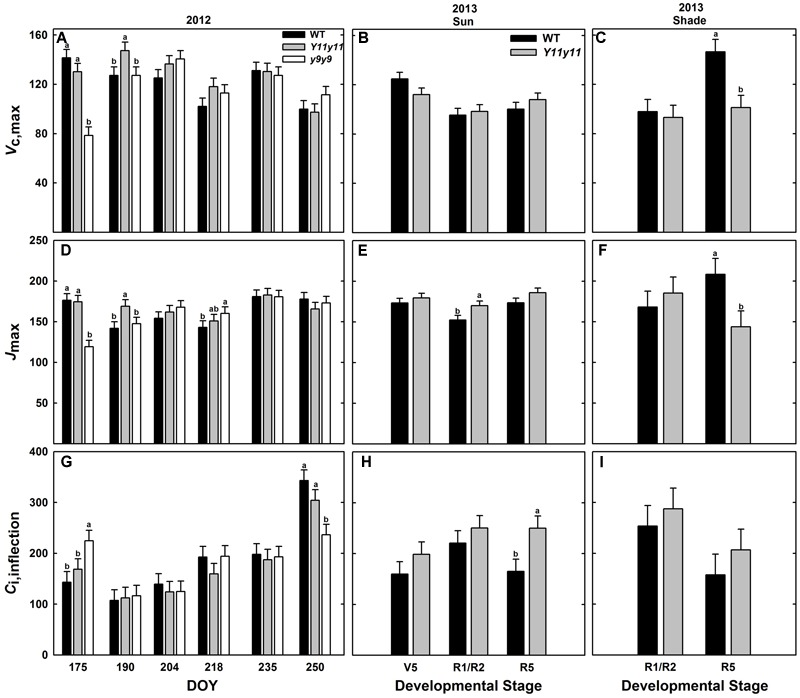
**Parameters from photosynthetic CO_2_ response (*A*/*C*_i_) curves across two growing seasons.** *V*_c,max_ **(A–C)**, *J*_max_ **(D–F)**, and *C*_i,inflection_ **(G–I)** from 2012 sun leaves **(A,D,G)**, 2013 sun leaves **(B,E,H)**, and 2013 shade leaves **(C,F,I)** are reported for WT (black), *Y11y11* (gray), and *y9y9* (white) soybean. Error bars represent standard errors (*n* = 3). Letters indicate significant differences within DOY or developmental stage (α = 0.1) when present.

Enhanced biochemical photosynthetic capacity, as measured by *A*/*C*_i_ curves, were transient in these specific mutants. Within sun leaf analyses, *Y11y11* had higher *V*_c,max_ compared to WT on DOY 190 in 2012 (**Figure 7A**). Conversely, *V*_c,max_ was reduced in *y9y9* on DOY 175 in 2012 (**Figure 7A**). This corresponded to a >85% reduction in *y9y9* chl content (**Figure 2C**) and a significant decrease in *A*_leaf_ (**Figure 5A**). There were no significant differences between WT and *Y11y11 V*_c,max_ in 2013 (**Figure 7B**). *J*_max_ was also slightly lower in *y9y9* sun leaves on DOY 175 (**Figure 7D**). However, *J*_max_ was greatest in *Y11y11* sun leaves on DOY 190 in 2012 (**Figure 7D**) and in the R1/R2 developmental stage in 2013 (**Figure 7E**). Both *V*_c,max_ and *J*_max_ showed similar relationships with chl content that declined steeply at chl contents less than 100 μmol m^-2^ while declining more gradually at chl contents greater than approximately 200 μmol m^-2^ (Supporting Information Figures S5B,C). Sun leaf *C*_i,inflection_ differed in *y9y9* compared to WT and *Y11y11* both early and late in the 2012 season (**Figure 7G**) with no significant effects occurring between WT and *Y11y11* in 2013 (Supporting Information Table S1 and **Figure 7H**). In 2013, WT and *Y11y11* shade leaf parameters did not differ in R1/R2, but *V*_c,max_ and *J*_max_ were significantly greater in WT compared to *Y11y11* in R5 (**Figures 7C,F,I**).

### *R*_d_ Was Significantly Lower in *Y11y11* than WT during 2013 Reproductive Stages

A significant genotype effect (*p* < 0.01) on *R*_d_ was evident in sun leaves during 2013 (Supporting Information Table S1). *Y11y11 R*_d_ in sun leaves was 13% lower than WT across the experiment (**Table 2** and Supporting Information Figure S6A). An apparent reduction of ∼12% was seen in *Y11y11* shade leaves (**Table 2** and Supporting Information Figure S6B), but shade leaf genotype effects on *R*_d_ were not significant (Supporting Information Table S1).

### Mutant Canopies Absorbed Less Light Early in the Season Despite Similar LAI

**FIGURE 8:**
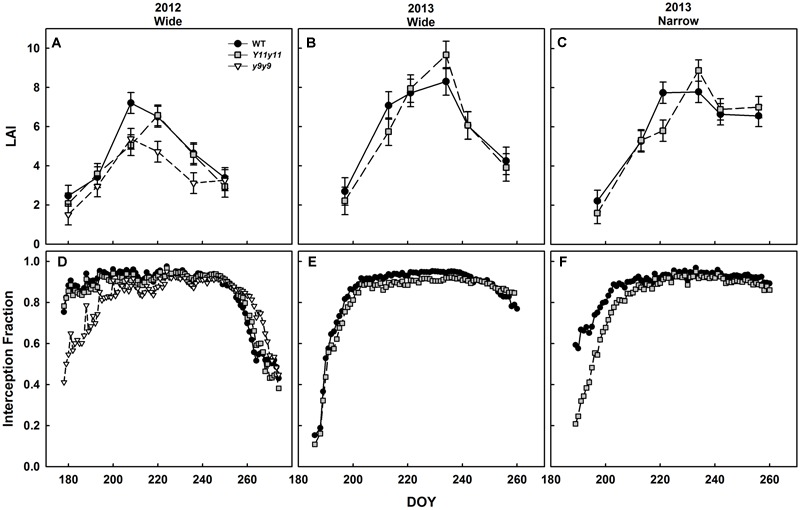
**Canopy leaf area index (LAI) and light interception fractions across two growing seasons.** LAI **(A–C)** in WT dark-green soybean (black circles) and two chl-deficient soybean mutants (*Y11y11* = gray squares; *y9y9* = white triangles) was calculated from biomass harvests in 2012 **(A)**, 2013 wide row spacing **(B)**, and 2013 narrow row spacing **(C)**. Light interception fraction of the canopy was also measured in 2012 **(D)** and 2013 wide **(E)** and narrow row spacing **(F)**. Error bars in **(A–C)** represent standard errors (*n* = 3) but were not included in **(D–F)** for clarity.

Canopy LAI was only significantly affected by chl reductions in 2012 (*p* < 0.05). In 2012, WT and *y9y9* reached peak LAI on DOY 208, on which day WT LAI was significantly greater than mutant LAI by 25–30% (**Figure 8A**). *Y11y11* did not reach peak LAI until DOY 220 (**Figure 8A**). *y9y9* LAI was also significantly lower than WT by ∼30% on DOY 220 and 236 (**Figure 8A**). There were no significant within-day differences between WT and *Y11y11* LAI in 2013 wide row widths (**Figure 8B**). Within-day differences between WT and *Y11y11* LAI in 2013 were only significant in the narrow row widths on DOY 221 (**Figure 8C**).

Light interception measurements in 2012 began when both the WT and *Y11y11* canopies had already reached interception fractions of ∼0.8 (**Figure 8D**). The *y9y9* canopy did not reach this fraction of light interception until approximately 2 weeks later but intercepted slightly more light at the end of the season (**Figure 8D**). Light interception measurements began relatively earlier in canopy development in 2013 and showed a slight lag in light interception by the *Y11y11* canopy as compared to WT in wide row spacing (**Figure 8E**) and a substantial lag in the narrow row spacing (**Figure 8F**).

### *A*_can_ Was Similar in WT and *Y11y11* Plots during the 2013 Season

**FIGURE 9:**
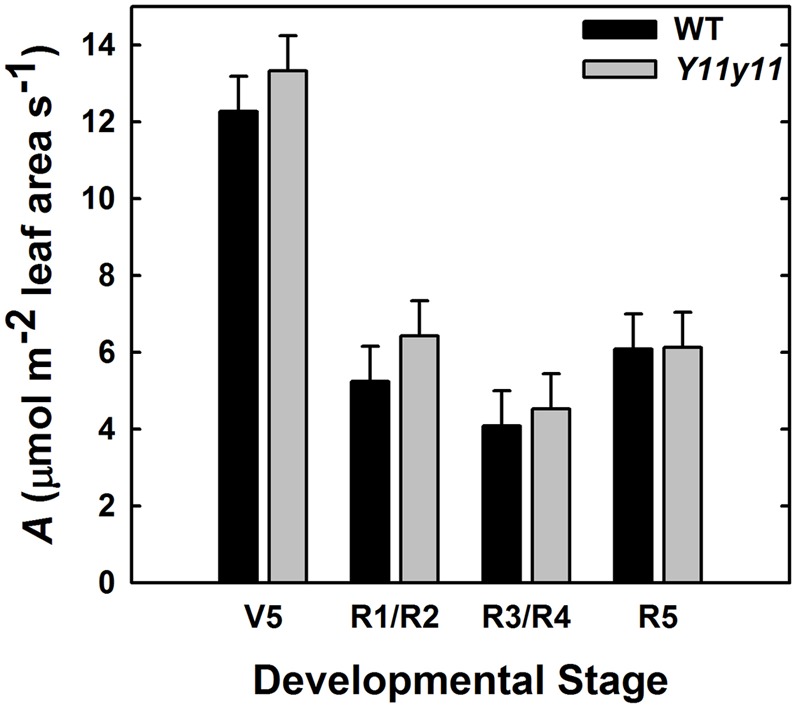
**Photosynthetic rate *(A)* per unit leaf area of the entire canopy across several developmental stages during the 2013 growing season.** *A*_can_ in WT (black) and *Y11y11* (gray) was measured using a chamber and reported after correcting for soil respiration and leaf area inside the chamber. Error bars represent the standard and errors (*n* = 3). There were no significant differences between values within developmental stages (α = 0.1).

*A*_can_ was calculated for each genotype based on CO_2_ drawdown rates that accounted for soil respiration and leaf area within an enclosed chamber. The WT and *Y11y11* canopies had similar *A*_can_ on a leaf area basis (*p* = 0.41; **Figure 9**). A significant DOY effect (*p* < 0.0001) most likely occurred because average photosynthetic rates on a leaf area basis decreased drastically after canopy closure (**Figures 8B,E**) as the area of shaded leaves increased in proportion to fully sunlit leaves in both genotypes (data not shown).

### Effects of Chl Reductions Were More Pronounced on 𝜀_i_ Compared to 𝜀_c_ or 𝜀_p_

𝜀_i_, 𝜀_c_, and 𝜀_p_ were calculated for WT, *Y11y11*, and *y9y9* in 2012 and WT and *Y11y11* in 2013, which also incorporated a row spacing treatment. In 2012, a significant decrease occurred in *y9y9* 𝜀_i_ compared to WT and *Y11y11*, and 𝜀_i_ was significantly lower in narrow rows of *Y11y11* compared to WT in 2013 (**Tables 4, 5**). In calculating 𝜀_c_, biomass was corrected for leaf, stem, and pod energy content. Pod energy, measured in 2013 only, differed between WT and *Y11y11* during late R5 (Supporting Information Table S2) and was 1 MJ kg^-1^ lower during R8 than the reported value from Amthor et al. (1994; Supporting Information Table S2). Although differences in 𝜀_c_ were not resolvable at α = 0.1 (**Table 4**), the percent reduction between WT and *Y11y11* 𝜀_c_ was less in the wide row spacing experiment of 2013 (3%) compared to 2012 (10%), and *Y11y11* 𝜀_c_ was almost 10% greater than WT 𝜀_c_ in the narrow row spacing of 2013 (**Table 5**). 𝜀_p_ was not significantly affected by genotype in either year or row spacing treatment (**Tables 4, 5**).

**Table 4:** ANOVA results of genotype effects on canopy parameters across two different years and row spacing treatment levels in 2013.

	2012		2013			
	0.38 m row space		0.38 m row space		0.19 m row space	
Parameter	*F*-value	*P*-value	*F*-value	*P*-value	*F*-value	*P*-value
𝜀_i_	8.81	<0.05	4.56	0.17	16.6	<0.1
𝜀_c_	0.87	0.43	0.47	0.50	0.79	0.38
𝜀_p_	2.72	0.18	1.85	0.31	4.40	0.17
Seed yield	8.77	<0.05	38.8	<0.05	1.15	0.40
Seed mass	2.78	0.18	784	<0.01	9.41	<0.1

*Effects of genotype on interception efficiency (𝜀_*i*_), conversion efficiency (𝜀_*c*_), partition efficiency (𝜀_*p*_), seed yield, and seed mass are indicated.*

**Table 5:** Parameter estimates of canopy level processes related to the Monteith equation (Monteith, 1977) and yield.

	2012			2013			
	0.38 m row space			0.38 m row space		0.19 m row space	
Parameter	WT	*Y11y11*	*y9y9*	WT	*Y11y11*	WT	*Y11y11*
Incident PAR (MJ m^-2^)	883	883	883	642	642	642	642
𝜀_i_	0.868a (0.034)	0.860a (0.034)	0.801b (0.034)	0.882a (0.008)	0.857a (0.008)	0.878a (0.012)	0.807b (0.012)
𝜀_c_	0.0226a (0.002) 12	0.0204a (0.001) 14	0.0198a (0.002) 14	0.0272a (0.003) 18	0.0263a (0.003) 18	0.0319a (0.002) 18	0.0330a (0.004) 18
𝜀_p_	0.476a (0.017)	0.490a (0.017)	0.518a (0.017)	0.416a (0.021)	0.421a (0.021)	0.495a (0.033)	0.542a (0.033)
Seed yield (g m^-2^)	184.4a (7.8)	157.2ab (7.8)	136.7b (7.8)	324.6a (11.5)	223.5b (11.5)	281.1a (42.9)	216.1a (42.9)
Seed mass (g (100 seeds)^-1^)	15.5a (0.40)	14.4ab (0.40)	14.2b (0.40)	15.0a (0.38)	13.0b (0.38)	15.5a (0.55)	13.2b (0.55)

*Estimates are across two growing seasons and two different row spacing treatment levels in 2013. Incident photosynthetically active radiation (PAR) is reported for the duration of the measurements. Interception efficiency (𝜀_*i*_), conversion efficiency (𝜀_*c*_), partition efficiency (𝜀_*p*_) are reported along with measured seed yield and mass per 100 seeds. Values within experiments with different letters represent significant differences at *p* < 0.1. The values in parentheses represent standard error of the regression slope for 𝜀_*c*_ and mean square error from ANOVA analyses in all other parameters. Sample size for determining 𝜀_*c*_ is indicated next to the standard errors and was *n* = 3 for all other parameters.*

### Reducing Chl Content Did Not Improve Yield in the Mutants

Overall yields were generally lower in 2012 compared to 2013 (**Table 5**), even though *S*_t_ was 30% greater in 2012 (**Table 1**). The lower yield is most likely due to a severe drought that occurred early in the 2012 growing season (**Figure 1C**). WT yield was significantly greater than *y9y9* yield in 2012 and *Y11y11* yield in 2013 wide row spacing (**Table 4**). Measured yields were not significantly different in the narrow row spacing of 2013 (**Table 5**). In addition, seed mass per 100 seeds was significantly reduced in *y9y9* during 2012 and *Y11y11* during 2013 (**Table 5**).

## Discussion

Reducing chl content in soybean was hypothesized to lead to an improved distribution of light in the canopy, resulting in benefits to leaf and canopy photosynthesis and therefore yield (Zhu et al., 2010; Ort et al., 2011; Drewry et al., 2014). This hypothesis was tested on two chl-deficient mutants that reportedly performed well in optimal field conditions in a previous study (Pettigrew et al., 1989). As the 1st year of the field study revealed severe limitations in the *y9y9* mutant, the 2nd year focused on comparison of the *Y11y11* mutant to the WT and by adding a narrow row spacing treatment that modeling predicted would advantage the light-green phenotype due to earlier canopy closure. Despite transient benefits to mutant leaf-level photosynthesis, no increases in canopy-level processes were evident during either growing season, both of which experienced drought conditions. While the results of this study confirmed neither our hypothesis nor the earlier published work in which the *Y11y11* mutant significantly outyielded the parental Clark cultivar, they do indicate that soybean, and likely many other crop plants, significantly overinvest in chl since a >50% chl reduction had little negative impact on biomass accumulation or yield. In addition, the small negative effects reduced chl did have on yield in our study were likely due to pleiotropic effects of the mutation. This outcome suggests that more sophisticated approaches for chl reduction, such as developmentally timed transgenic technology, may promote an opportunity to reinvest nitrogen and energy resources saved in chl reduction into increasing the biochemical photosynthetic capacity, leading to increased yield.

Although the *Y11y11* mutant demonstrated the potential to surpass WT in light use efficiency and *A*_sat_, the improvements were transient. The increases in light use efficiency corresponded with a narrow range of chl reductions (**Table 3** and **Figures 2C,D, 6**), which may support the hypothesis that an optimal chl content exists and is lower than current levels (Ort et al., 2011). Benefits to *A*_leaf_ were seen only when chl content in *Y11y11* was reduced to 30–40% of WT (**Figures 2C,D**), which corresponded to a 22% reduction in leaf_abs_ (**Figure 3**) that is less than the reduction predicted by Beer’s law (Slattery et al., 2016). As chl content increased in the mutant throughout the growing season (**Figures 2C,D**) and the percent difference between mutant and WT leaf_abs_ decreased (**Figure 3**), benefits at the leaf level became too small to resolve (**Figure 6**). In other species, chl contents correlated with increased *A*_leaf_ ranged from 30% in tobacco (Edwards et al., 1993) to 60% in maize (Edwards et al., 1993) and cowpea (Habash et al., 1994) as compared to the dark-green WT, suggesting that optimal chl content may be species specific.

Improved leaf light use efficiency is the anticipated result of improved light distribution within the low chl leaf. In dark-green leaves, the majority of light absorption occurs in the uppermost adaxial chloroplasts, causing light limitation in lower chloroplasts (Vogelmann and Evans, 2002; Evans and Vogelmann, 2003; Slattery et al., 2016). This has been shown to create a within-leaf gradient in photosynthesis (Evans and Vogelmann, 2003) as well as photoinhibition (Oguchi et al., 2011) that decreases with depth in the leaf and limits overall photosynthetic efficiency. Light sheet microscopy analyses revealed more gradual PPFD attenuation in light-green soybean leaves compared to dark-green leaves. This resulted in significantly more red and blue light reaching the spongy mesophyll chloroplasts of the light-green leaves, which correlated with greater photosynthetic light use efficiency (Slattery et al., 2016). Thus, if chloroplasts within the leaf mimic leaves within a canopy, then decreasing chl content may have ameliorated the large disparity of light availability in the lower leaf to increase photosynthetic light use efficiency.

However, other attributes of the chl mutants may have also played roles in altering photosynthetic performance. SLW was reduced in both mutants (**Figures 2A,B**). Changes in SLW are due to changes in either leaf thickness or leaf density, both of which can affect photosynthesis (Niinemets, 1999). Although leaf thickness was not directly measured in this study, leaf thickness did not change in a previous study on the same soybean genotypes, even when SLW was significantly different (Slattery et al., 2016). Therefore, changes in SLW were almost certainly due to reduced leaf density in the chl mutants, which can increase the proportion of intercellular space and thus improve CO_2_ diffusion throughout the leaf (Niinemets, 1999). In addition, chl deficiency in these mutants was accompanied by increased *g*_s_, which would increase carbon supply to the inner leaf and further confound deciphering the chl reduction impact on *A*_leaf_. A similar increase in *g*_s_ was seen in rice chl mutants that exhibited greater rates of *A*_leaf_ in non-limiting environmental conditions (Gu et al., 2017). Those authors hypothesized that the increase in *g*_s_ may have been due to changes in vein structure in the rice mutants (Gu et al., 2017). More analyses are needed to separate the effects of reduced chl, increased *g*_s_, and altered leaf anatomy on *A*_leaf_ improvements or the lack thereof.

A decline in photosynthetic efficiency and capacity in *y9y9* was correlated with severe reductions in chl. Despite greater *g*_s_ and lower SLW, which was evident in *Y11y11* as well, the *y9y9* mutant displayed reduced *A*_sat_ and light use efficiency compared to the WT (**Figures 4A, 5A, 6, 7A** and **Table 3**) when chl was reduced by more than 85% compared to the WT (**Figure 2C**). Similar responses to severe reductions in chl content (>80%) seen in *y9y9* have been reported in *Y11y11* when grown in controlled environment chambers at lower than field light levels (Xu et al., 1993; Slattery et al., 2016). These two mutants are characterized by higher chl *a*/*b* ratios due to greatly reduced PSII-associated light harvesting complexes (LHCII) and to some extent PSI-associated light harvesting complexes (LHCI; Ghirardi and Melis, 1988). Theoretically, severe deficiencies in LHCII could have negative effects at the leaf level, including greater levels of photoinhibition (Leverenz et al., 1992; Xu et al., 1993), reduced photoprotective capacity (Ort, 2001), and lowered connectivity among PSII centers and therefore lower quantum yield at low light (Allen and Forsberg, 2001). In this study, extreme reductions in chl coupled with large reductions in carotenoids (>40%; **Table 2**) were correlated with impaired photoprotective mechanisms such as *NPQ* (**Figure 6**). ϕ*CO*_2_ was also significantly reduced in *y9y9* (**Tables 2, 3**). Thus, these data suggest improving leaf photosynthetic efficiency through lowering chl *a* and chl *b* concentrations proportionally, but not to an extent that impairs photosynthetic and photoprotective capacity.

Despite transient leaf-level improvements in photosynthesis and photosynthetic efficiency, chl reductions only significantly affected the canopy parameter of 𝜀_i_. Although no improvements in *A*_can_ or 𝜀_c_ were evident in the chl-deficient soybean, the fact that there was also no decline in these parameters with a >50% reduction in chl suggests that soybean significantly overinvest in chl. This is consistent with a recent study that showed soybean also overinvest in LAI to the detriment of canopy productivity (Srinivasan et al., 2016). However, less pigment led to significant depressions in 𝜀_i_ in *y9y9* in 2012 and *Y11y11* in 2013 narrow row widths (**Tables 4, 5**), mostly due to reduced light interception by the mutants early in the season, even when LAI was similar (**Figure 8**). The mutant chl content in this study was the lowest early in the season (**Figures 2C,D**), which would be expected to both increase transmission to the soil and increase the proportion of reflectance not reabsorbed by upper canopy leaves during early growth. As LAI increased, chl content also increased in the mutants, which in turn would be expected to limit light penetration to deeper layers in the canopy. An ideal situation for maximizing light absorption early in the season would be normal chl content until the canopy has closed (Melis, 2009). This would suppress weed vigor through increased competitiveness, which is reasoned to be why plants evolved with much higher chl content than is needed to maximize photosynthesis (Donald, 1968). As LAI increases, decreasing chl biosynthesis in new leaves would alleviate oversaturation in times of high light and allow greater transmittance to the lower canopy. However, light reflection and thus loss of canopy absorbance will also increase at the top of the canopy; thus, a more light-use efficient canopy may not necessarily lead to an increase in *A*_can_ from chl reductions alone.

Reduced pigmentation was hypothesized to benefit leaf water use along with canopy 𝜀_c_, but the greater *g*_s_ that accompanied chl reductions in these mutants likely prevented these effects. Increased albedo was associated with lower *T*_leaf_ in the mutants as expected (**Figures 5G,H**), but cooler *T*_leaf_ may have been, at least in part, an effect of greater *g*_s_ and therefore cooling via transpiration in the mutants (**Figures 5E,F**). Greater *g*_s_ was most likely a result of the mutations causing chl deficiency. A recent study identified a mutation in the magnesium chelatase subunit-I gene (CHLI) as the cause of the light-green phenotype of *Y11y11* (Campbell et al., 2015). In *A. thaliana*, both CHLI and CHLH mutants have demonstrated ABA-insensitivity (Tsuzuki et al., 2011; Du et al., 2012) that is independent of chl biosynthesis (Du et al., 2012). Whether the greater *g*_s_ in *y9y9* that occurred in this study is also due to ABA-insensitivity is unknown since the mutation has not yet been identified, but greater *g*_s_ has been reported in *y9y9* compared to WT regardless of water stress (Luquez et al., 1997). The increase in *g*_s_ resulted in lower mutant iWUE (**Figures 5C,D**) and integrated canopy WUE (Supporting Information Figure S4). Lower canopy WUE in the mutants would likely result in greater soil moisture depletion (Hussain et al., 2013) and therefore greater susceptibility to drought, which occurred during both of the growing seasons (**Figures 1C,D**). Drought stress significantly reduces 𝜀_c_ (Slattery et al., 2013), and the greater susceptibility to drought in the mutants may have dampened even small benefits of reduced chl on 𝜀_c_ and yield. In the previous study where *A*_can_ and yields were greater in *Y11y11* compared to WT, water was not limiting due to irrigation (Pettigrew et al., 1989), suggesting that these specific mutations causing chl deficiency may limit productivity in times of even moderate water stress. Although rice chl mutants with greater *g*_s_ resulted in higher yields when grown at a higher planting density, the authors do not report any water limitations. Further assessments of chl-deficient crops may need to incorporate tests of efficiency and productivity in non-optimal field conditions while maintaining or improving WUE.

The results of this study suggest that soybean overinvest in chl; thus, chl reduction represents an opportunity to reinvest nitrogen from pigment-proteins into rate limiting photosynthetic enzymes that could increase photosynthetic capacity (i.e., *V*_c,max_ and *J*_max_). However, nitrogen reallocation was not realized to any significant extent in these mutants. For example, a study by Evans and Poorter (2001) showed that 12.9% of leaf organic nitrogen was associated with pigment-protein complexes and 21.6% was associated with Rubisco in plants grown at high light. Therefore, if pigment-proteins were reduced by 50% and all of the nitrogen associated with pigment-proteins was reallocated to Rubisco, carboxylation capacity could potentially increase by up to 30%. However, across both seasons, *Y11y11 V*_c,max_ increased <5% compared to WT (**Table 2**). *J*_max_ only increased by 3% in 2012 and 7% in 2013 in *Y11y11* compared to WT (**Table 2**). Therefore, further intervention would be required to redirect nitrogen savings from reduced investment in pigment-proteins in low-chl plants to the most beneficial targets for increasing photosynthesis (Zhu et al., 2007).

## Conclusion

This study demonstrated early season benefits of reduced chl content on leaf photosynthetic efficiency and capacity. However, the effects of reduced chl were confounded with leaf properties and greater *g*_s_ in this study and a similar study in rice. Further analyses will be needed to parse out the impacts of chl reductions on leaf structure, *g*_s_, and *A*_leaf_. The mutants used in this study, although relatively robust in optimal field conditions, captured less light early in the growing season and used water less efficiently, which may have impaired the effects of reduced chl during the drought conditions experienced in both growing seasons. Despite the pleiotropic effects of the mutations, limitations to biomass accumulation and yield were minimal, signifying an overinvestment in chl in dark-green soybean.

As evident from these results, the methods of obtaining and maintaining optimal chl concentrations require further consideration. Optimizing chl concentration within the canopy and throughout the season is required to reduce wasted light early in the season via transmission to the soil and later in the season as reflected light from the top of the canopy while maintaining a more even light distribution within the canopy. Additionally, it is crucial to identify and utilize targets that eliminate the pleiotropic effects, such as greater water loss and photooxidative effects, that can accompany many chl biosynthesis mutations. Lastly, nitrogen reinvestment will need optimization to maximize nitrogen use efficiency in low-chl plants. This suggests that directed approaches, such as transgenic technology, are required for greater benefits of reduced chl on *A*_can_, 𝜀_c_, and yield.

## Author Contributions

All authors participated conceiving and designing the research. RS, AV, and CB performed the research. RS and AV analyzed the data. RS and DO wrote the article, which was reviewed by all other authors.

## Conflict of Interest Statement

The authors declare that the research was conducted in the absence of any commercial or financial relationships that could be construed as a potential conflict of interest.

## References

[B1] Ainsworth E. A. Ort D. R. (2010). How do we improve crop production in a warming world? *Plant Physiol.* 154 526–530. 10.1104/pp.110.161349 20921178 PMC2949002

[B2] Allen J. F. Forsberg J. (2001). Molecular recognition in thylakoid structure and function. *Trends Plant Sci.* 6 317–326. 10.1016/S1360-1385(01)02010-6 11435171

[B3] Amthor J. S. Mitchell R. J. Runion G. B. Rogers H. H. Prior S. A. Wood C. W. (1994). Energy content, construction cost and phytomass accumulation of *Glycine max* (L.) Merr. and *Sorghum bicolor* (L.) Moench grown in elevated CO_2_ in the field. *New Phytol.* 128 443–450. 10.1111/j.1469-8137.1994.tb02990.x 33874580

[B4] Angel J. (2009). *The Water and Atmospheric Resources Monitoring Program.* Urbana, IL: University of Illinois at Urbana-Champaign.

[B5] Austin R. Bingham J. Blackwell R. Evans L. Ford M. Morgan C. (1980). Genetic improvements in winter wheat yields since 1900 and associated physiological changes. *J. Agric. Sci.* 94 675–689. 10.1017/S0021859600028665

[B6] Baker N. R. (2008). Chlorophyll fluorescence: a probe of photosynthesis in vivo. *Annu. Rev. Plant Biol.* 59 89–113. 10.1146/annurev.arplant.59.032607.092759 18444897

[B7] Baldocchi D. D. Verma S. B. Rosenberg N. J. (1985). Water use efficiency in a soybean field: influence of plant water stress. *Agric. For. Meteorol.* 34 53–65. 10.1016/0168-1923(85)90054-1

[B8] Benedict C. R. McCree K. J. Kohel R. J. (1972). High photosynthetic rate of a chlorophyll mutant of cotton. *Plant Physiol.* 49 968–971. 10.1104/pp.49.6.968 16658093 PMC366089

[B9] Bugbee B. G. Monje O. (1992). The limits of crop productivity. *Bioscience* 42 494–502. 10.2307/1311879 11537403

[B10] Campbell B. W. Mani D. Curtin S. J. Slattery R. A. Michno J.-M. Ort D. R. (2015). Identical substitutions in magnesium chelatase paralogs result in chlorophyll deficient soybean mutants. *G3 (Bethesda)* 5 123–131. 10.1534/g3.114.015255 PMC4291463 25452420

[B11] Campbell G. S. Norman J. M. (1998). “The light environment of plant canopies,” in *An Introduction to Environmental Biophysics* (New York, NY: Springer-Verlag) 247–278. 10.1007/978-1-4612-1626-1_15

[B12] Dermody O. Long S. P. McConnaughay K. DeLucia E. H. (2008). How do elevated CO_2_ and O_3_ affect the interception and utilization of radiation by a soybean canopy? *Glob. Chang. Biol.* 14 556–564. 10.1111/j.1365-2486.2007.01502.x

[B13] Donald C. M. (1968). The breeding of crop ideotypes. *Euphytica* 17 385–403. 10.1007/BF00056241

[B14] Drewry D. T. Kumar P. Long S. P. (2014). Simultaneous improvement in productivity, water use, and albedo through crop structural modification. *Glob. Chang. Biol.* 20 1955–1967. 10.1111/gcb.12567 24700722

[B15] Du S.-Y. Zhang X.-F. Lu Z. Xin Q. Wu Z. Jiang T. (2012). Roles of the different components of magnesium chelatase in abscisic acid signal transduction. *Plant Mol. Biol.* 80 519–537. 10.1007/s11103-012-9965-3 23011401 PMC3472068

[B16] Edwards G. E. Johnson E. Lal A. Krall J. P. (1993). Quantum yields of photosystem II and photosynthesis in an aurea mutant of tobacco (C_3_) and an oil yellow mutant of maize (C_4_) which have high capacities for photosynthesis despite low chlorophyll contents. *Plant Cell Physiol.* 34 1205–1212.

[B17] Eskins K. Delmastro D. Harris L. (1983). A comparison of pigment-protein complexes among normal, chlorophyll-deficient and senescent soybean genotypes. *Plant Physiol.* 73 51–55. 10.1104/pp.73.1.51 16663184 PMC1066405

[B18] Eskins K. Harris L. Bernard R. L. (1981). Genetic control of chloroplast pigment development in soybeans as a function of leaf and plant maturity. *Plant Physiol.* 67 759–762. 10.1104/pp.67.4.759 16661750 PMC425768

[B19] Estill K. Delaney R. H. Smith W. K. Ditterline R. L. (1991). Water relations and productivity of alfalfa leaf chlorophyll variants. *Crop Sci.* 31 1229–1233. 10.2135/cropsci1991.0011183X003100050030x

[B20] Evans J. R. Poorter H. (2001). Photosynthetic acclimation of plants to growth irradiance: the relative importance of specific leaf area and nitrogen partitioning in maximizing carbon gain. *Plant Cell Environ.* 24 755–767. 10.1046/j.1365-3040.2001.00724.x

[B21] Evans J. R. Vogelmann T. C. (2003). Profiles of 14C fixation through spinach leaves in relation to light absorption and photosynthetic capacity. *Plant Cell Environ.* 26 547–560. 10.1046/j.1365-3040.2003.00985.x

[B22] Evans L. T. (1993). *Crop Evolution, Adaptation, and Yield.* Cambridge: Cambridge University Press.

[B23] Evans L. T. Fischer R. A. (1999). Yield potential: its definition, measurement, and significance. *Crop Sci.* 39 1544–1551. 10.2135/cropsci1999.3961544x

[B24] FAO (2012). *Food and Agricultural Commodities Production.* Rome: FAO.

[B25] Farquhar G. D. Richards R. A. (1984). Isotopic composition of plant carbon correlates with water-use efficiency of wheat genotypes. *Aust. J. Plant Physiol.* 11 539–552. 10.1071/PP9840539

[B26] Fehr W. Caviness C. Burmood D. T. Pennington J. S. (1971). Stage of development descriptions for soybeans, *Glycine max* (L.) Merr. *Crop Sci.* 11 929–931. 10.2135/cropsci1971.0011183X001100060051x

[B27] Gamon J. A. Pearcy R. W. (1989). Leaf movement, stress avoidance and photosynthesis in *Vitis californica*. *Oecologia* 79 475–481. 10.1007/BF00378664 28313481

[B28] Ghirardi M. L. Melis A. (1988). Chlorophyll *b* deficiency in soybean mutants. I. Effects on photosystem stoichiometry and chlorophyll antenna size. *Biochim. Biophys. Acta* 932 130–137. 10.1016/0005-2728(88)90147-8

[B29] Gillespie K. M. Xu F. Richter K. T. McGrath J. M. Markelz R. J. C. Ort D. R. (2012). Greater antioxidant and respiratory metabolism in field-grown soybean exposed to elevated O_3_ under both ambient and elevated CO_2_. *Plant Cell Environ.* 35 169–184. 10.1111/j.1365-3040.2011.02427.x 21923758

[B30] Gu J. Zhou Z. Li Z. Chen Y. Wang Z. Zhang H. (2017). Rice (*Oryza sativa* L.) with reduced chlorophyll content exhibit higher photosynthetic rate and efficiency, improved canopy light distribution, and greater yields than normally pigmented plants. *Field Crop Res.* 200 58–70. 10.1016/j.fcr.2016.10.008

[B31] Habash D. Z. Genty B. Baker N. R. (1994). The consequences of chlorophyll deficiency for photosynthetic light use efficiency in a single nuclear gene mutation of cowpea. *Photosynth. Res.* 42 17–25. 10.1007/BF00019054 24307464

[B32] Hay R. (1995). Harvest index: a review of its use in plant breeding and crop physiology. *Ann. Appl. Biol.* 126 197–216. 10.1111/j.1744-7348.1995.tb05015.x

[B33] Hay R. Porter J. (2006). *The Physiology of Crop Yield.* Oxford: Blackwell Publishing.

[B34] Highkin H. R. Boardman N. K. Goodchild D. J. (1969). Photosynthetic studies on a pea mutant deficient in chlorophyll. *Plant Physiol.* 44 1310–1320. 10.1104/pp.44.9.1310 16657204 PMC396261

[B35] Hussain M. Z. Vanloocke A. Siebers M. H. Ruiz-Vera U. M. Markelz R. J. C. Leakey A. D. B. (2013). Future carbon dioxide concentration decreases canopy evapotranspiration and soil water depletion by field-grown maize. *Glob. Chang. Biol.* 19 1572–1584. 10.1111/gcb.12155 23505040

[B36] Khush G. S. (1995). Breaking the yield frontier of rice. *GeoJournal* 35 329–332. 10.1007/BF00989140

[B37] Kirst H. Gabilly S. T. Niyogi K. K. Lemaux P. G. Melis A. (2017). Photosynthetic antenna engineering to improve crop yields. *Planta* 10.1007/s00425-017-2659-y [Epub ahead of print]. 28188423

[B38] Koester R. P. Skoneczka J. A. Cary T. R. Diers B. W. Ainsworth E. A. (2014). Historical gains in soybean (*Glycine max* Merr.) seed yield are driven by linear increases in light interception, energy conversion, and partitioning efficiencies. *J. Exp. Bot.* 65 3311–3321. 10.1093/jxb/eru187 24790116 PMC4071847

[B39] Kok B. (1948). A critical consideration of the quantum yield of *Chlorella* photosynthesis. *Enzymologia* 13 1–56.

[B40] Kosourov S. N. Ghirardi M. L. Seibert M. (2011). A truncated antenna mutant of *Chlamydomonas reinhardtii* can produce more hydrogen than the parental strain. *Int. J. Hydrogen Energy* 36 2044–2048. 10.1016/j.ijhydene.2010.10.041

[B41] Leverenz J. W. Oqtaist G. Wingsle G. (1992). Photosynthesis and photointiibition in leaves of chlorophyll b-less barley in relation to absorbed light. *Physiol. Plant.* 85 495–502. 10.1111/j.1399-3054.1992.tb05817.x

[B42] Li Y. Ren B. Gao L. Ding L. Jiang D. Xu X. (2013). Less chlorophyll does not necessarily restrain light capture ability and photosynthesis in a chlorophyll-deficient rice mutant. *J. Agron. Crop Sci.* 199 49–56. 10.1111/j.1439-037X.2012.00519.x

[B43] Lichtenthaler H. (1987). Chlorophylls and carotenoids: pigments of photosynthetic biomembranes. *Methods Enzymol.* 148 350–382. 10.1016/0076-6879(87)48036-1

[B44] Lobell D. B. Cassman K. G. Field C. B. (2009). Crop yield gaps: their importance, magnitudes, and causes. *Annu. Rev. Environ. Resour.* 34 179–204. 10.1146/annurev.environ.041008.093740

[B45] Long S. P. Bernacchi C. J. (2003). Gas exchange measurements, what can they tell us about the underlying limitations to photosynthesis? Procedures and sources of error. *J. Exp. Bot.* 54 2393–2401. 10.1093/jxb/erg262 14512377

[B46] Long S. P. Zhu X.-G. Naidu S. L. Ort D. R. (2006). Can improvement in photosynthesis increase crop yields? *Plant Cell Environ.* 29 315–330. 10.1111/j.1365-3040.2005.01493.x 17080588

[B47] Luquez V. M. Guiamet J. J. Montaldi E. R. (1997). Net photosynthetic and transpiration rates in a chlorophyll-deficient isoline of soybean under well-watered and drought conditions. *Photosynthetica* 34 125–131. 10.1023/A:1006824120129

[B48] Melis A. (1999). Photosystem-II damage and repair cycle in chloroplasts: what modulates the rate of photodamage in vivo? *Trends Plant Sci.* 4 130–135. 10.1016/S1360-1385(99)01387-4 10322546

[B49] Melis A. (2009). Solar energy conversion efficiencies in photosynthesis: minimizing the chlorophyll antennae to maximize efficiency. *Plant Sci.* 177 272–280. 10.1016/j.plantsci.2009.06.005

[B50] Mitra M. Melis A. (2008). Optical properties of microalgae for enhanced biofuels production. *Opt. Express* 16 21807–21820. 10.1364/OE.16.021807 19104614

[B51] Monteith J. L. (1965). Radiation and crops. *Exp. Agric.* 1 241–251. 10.1017/S0014479700021529

[B52] Monteith J. L. (1972). Solar radiation and productivity in tropical ecosystems. *J. Appl. Ecol.* 9 747–766. 10.2307/2401901

[B53] Monteith J. L. (1977). Climate and the efficiency of crop production in Britain. *Philos. Trans. R. Soc. Lond. B Biol. Sci.* 281 277–294. 10.1098/rstb.1977.0140

[B54] Niinemets Ü (1999). Components of leaf dry mass per area - thickness and density - alter leaf photosynthetic capacity in reverse directions in woody plants. *New Phytol.* 144 35–47. 10.1046/j.1469-8137.1999.00466.x

[B55] Niyogi K. K. (1999). Photoprotection revisited: genetic and molecular approaches. *Annu. Rev. Plant Physiol. Plant Mol. Biol.* 50 333–359. 10.1146/annurev.arplant.50.1.333 15012213

[B56] Oguchi R. Douwstra P. Fujita T. Chow W. S. Terashima I. (2011). Intra-leaf gradients of photoinhibition induced by different color lights: implications for the dual mechanisms of photoinhibition and for the application of conventional chlorophyll fluorometers. *New Phytol.* 191 146–159. 10.1111/j.1469-8137.2011.03669.x 21418065

[B57] Ort D. R. (2001). When there is too much light. *Plant Physiol.* 125 29–32. 10.1104/pp.125.1.29 11154289 PMC1539318

[B58] Ort D. R. Long S. P. (2014). Limits on yields in the Corn Belt. *Science* 344 484–485. 10.1126/science.1253884 24786071

[B59] Ort D. R. Merchant S. S. Alric J. Barkan A. Blankenship R. E. Bock R. (2015). Redesigning photosynthesis to sustainably meet global food and bioenergy demand. *Proc. Natl. Acad. Sci. U.S.A.* 112 8529–8536. 10.1073/pnas.1424031112 26124102 PMC4507207

[B60] Ort D. R. Zhu X. Melis A. (2011). Optimizing antenna size to maximize photosynthetic efficiency. *Plant Physiol.* 155 79–85. 10.1104/pp.110.165886 21078863 PMC3014228

[B61] Pettigrew W. T. Hesketh D. Peters D. B. Woolley T. (1989). Characterization of canopy photosynthesis of chlorophyll-deficient soybean isolines. *Crop Sci.* 29 1024–1028. 10.2135/cropsci1989.0011183X002900040040x

[B62] Polle J. E. W. Kanakagiri S. Jin E. Masuda T. Melis A. (2002). Truncated chlorophyll antenna size of the photosystems–a practical method to improve microalgal productivity and hydrogen production in mass culture. *Int. J. Hydrogen Energy* 27 1257–1264. 10.1016/S0360-3199(02)00116-7

[B63] Porra R. Thompson W. Kriedemann P. (1989). Determination of accurate extinctions coefficients and simultaneous equations for assaying chlorophylls *a* and *b* extracted with four different solvents: verification of the concentration of chlorophyll standards by atomic absorption spectrosc. *Biochim. Biophys. Acta* 975 384–394. 10.1016/S0005-2728(89)80347-0

[B64] Prasad P. V. V. Boote K. J. Allen L. H. Jr. Sheehy J. E. Thomas J. M. G. (2006). Species, ecotype and cultivar differences in spikelet fertility and harvest index of rice in response to high temperature stress. *Field Crop Res.* 95 398–411. 10.1016/j.fcr.2005.04.008

[B65] Prater M. R. Obrist D. Arnone J. A. DeLucia E. H. (2006). Net carbon exchange and evapotranspiration in postfire and intact sagebrush communities in the Great Basin. *Oecologia* 146 595–607. 10.1007/s00442-005-0231-0 16151860

[B66] Rogers A. Allen D. J. Davey P. A. Morgan P. B. Ainsworth E. A. Bernacchi C. J. (2004). Leaf photosynthesis and carbohydrate dynamics of soybeans grown throughout their life-cycle under Free-Air Carbon dioxide Enrichment. *Plant Cell Environ.* 27 449–458. 10.1111/j.1365-3040.2004.01163.x

[B67] Sinclair T. R. (1998). Historical changes in harvest index and crop nitrogen accumulation. *Crop Sci.* 38 638–643. 10.2135/cropsci1998.0011183X003800030002x

[B68] Slattery R. A. Ainsworth E. A. Ort D. R. (2013). A meta-analysis of responses of canopy photosynthetic conversion efficiency to environmental factors reveals major causes of yield gap. *J. Exp. Bot.* 64 3723–3733. 10.1093/jxb/ert207 23873996 PMC3745731

[B69] Slattery R. A. Grennan A. K. Sivaguru M. Sozzani R. Ort D. R. (2016). Light sheet microscopy reveals more gradual light attenuation in light-green versus dark-green soybean leaves. *J. Exp. Bot.* 67 4697–4709. 10.1093/jxb/erw246 27329746 PMC4973739

[B70] Slattery R. A. Ort D. R. (2015). Photosynthetic energy conversion efficiency: setting a baseline for gauging future improvements in important food and biofuel crops. *Plant Physiol.* 168 383–392. 10.1104/pp.15.00066 25829463 PMC4453776

[B71] Smil V. (1999). Crop residues: agriculture’s largest harvest - crop residues incorporate more than half of the world’s agricultural phytomass. *Bioscience* 49 299–308. 10.2307/1313613

[B72] Srinivasan V. Kumar P. Long S. P. (2016). Decreasing, not increasing, leaf area will raise crop yields under global atmospheric change. *Glob. Chang. Biol.* 23 1626–1635. 10.1111/gcb.13526 27860122 PMC5347850

[B73] Tsuzuki T. Takahashi K. Inoue S. Okigaki Y. Tomiyama M. Hossain M. A. (2011). Mg-chelatase H subunit affects ABA signaling in stomatal guard cells, but is not an ABA receptor in *Arabidopsis thaliana*. *J. Plant Res.* 124 527–538. 10.1007/s10265-011-0426-x 21562844 PMC3129500

[B74] Vogelmann T. C. Evans J. R. (2002). Profiles of light absorption and chlorophyll within spinach. *Plant Cell Environ.* 25 1313–1323. 10.1046/j.1365-3040.2002.00910.x

[B75] Xu D. Chen X. M. Zhang L. X. Wang R. F. Hesketh J. D. (1993). Leaf photosynthesis and chlorophyll fluorescence in a chlorophyll-deficient soybean mutant. *Photosynthetica* 29 103–112.

[B76] Zhu X.-G. de Sturler E. Long S. P. (2007). Optimizing the distribution of resources between enzymes of carbon metabolism can dramatically increase photosynthetic rate: a numerical simulation using an evolutionary algorithm. *Plant Physiol.* 145 513–526. 10.1104/pp.107.103713 17720759 PMC2048738

[B77] Zhu X.-G. Long S. P. Ort D. R. (2008). What is the maximum efficiency with which photosynthesis can convert solar energy into biomass? *Curr. Opin. Biotechnol.* 19 153–159. 10.1016/j.copbio.2008.02.004 18374559

[B78] Zhu X.-G. Long S. P. Ort D. R. (2010). Improving photosynthetic efficiency for greater yield. *Annu. Rev. Plant Biol.* 61 235–261. 10.1146/annurev-arplant-042809-112206 20192734

