# Supplementary material for: Photosynthesis, Light Use Efficiency, and Yield of Reduced-Chlorophyll Soybean Mutants in Field Conditions
Source: Front Plant Sci. 2017 Apr 18;8:549. doi: 10.3389/fpls.2017.00549 (PMC5394119; doi:10.3389/fpls.2017.00549)
Supplement: Supplementary file 1 [file Data_Sheet_1.docx]

**Supporting Information Table S1**. Analysis of variance results for dark and light green soybean variables from two growing seasons and two leaf levels. Main effects were genotype and day of year (DOY). Results are reported for variables related to physical leaf properties, diurnal measurements, photosynthetic response curves, and respiration rates. Level of significance is indicated with ns=non-significant at p>0.1. Dashes indicate effects not measured.

|  |  | 2012 | | | 2013 | | |
| --- | --- | --- | --- | --- | --- | --- | --- |
| Leaf | Parameter | Genotype | DOY | Genotype*DOY | Genotype | DOY | Genotype*DOY |
| Sun | SLW | <0.0001 | <0.0001 | <0.0001 | <0.0001 | <0.0001 | ns |
|  | Chl content | <0.0001 | <0.0001 | <0.0001 | <0.0001 | <0.0001 | <0.01 |
|  | Chl *a*/*b* | <0.0001 | <0.0001 | <0.0001 | <0.0001 | <0.001 | 0.10 |
|  | Carotenoids | <0.0001 | <0.0001 | <0.01 | <0.0001 | <0.0001 | <0.05 |
|  | Leaf_abs_ | - | - | - | <0.0001 | <0.0001 | <0.05 |
|  | *A*′ | <0.0001 | <0.0001 | <0.0001 | ns | <0.0001 | <0.05 |
|  | *A*_leaf_ | <0.01 | <0.0001 | <0.05 | ns | <0.0001 | ns |
|  | *g*_s_ | <0.01 | <0.0001 | <0.05 | <0.0001 | <0.0001 | <0.01 |
|  | iWUE | <0.01 | <0.0001 | <0.01 | <0.0001 | <0.0001 | <0.001 |
|  | *T*_leaf_ | <0.0001 | <0.0001 | <0.01 | <0.0001 | <0.0001 | ns |
|  | *V*_c,max_ | ns | <0.0001 | <0.0001 | ns | <0.01 | ns |
|  | *J*_max_ | 0.09 | <0.0001 | <0.0001 | <0.01 | <0.05 | ns |
|  | *C*_i, inflection_ | ns | <0.0001 | <0.01 | ns | 0.010 | ns |
|  | *A*_sat_ | <0.05 | - | - | <0.01 | <0.0001 | ns |
|  | *ϕCO_2_* | <0.01 | - | - | <0.05 | <0.0001 | <0.1 |
|  | *R*_d_ | - | - | - | <0.01 | ns | ns |
|  |  |  |  |  |  |  |  |
| Shade | Chl content | <0.001 | - | - | <0.01 | - | - |
|  | Chl *a*/*b* | <0.0001 | - | - | 0.09 | - | - |
|  | *A*′ | ns | - | - | - | - | - |
|  | *V*_c,max_ | - | - | - | <0.05 | <0.05 | ns |
|  | *J*_max_ | - | - | - | ns | ns | 0.07 |
|  | *C*_i, inflection_ | - | - | - | 0.10 | 0.09 | ns |
|  | *A*_sat_ | - | - | - | ns | <0.05 | ns |
|  | *ϕCO_2_* | - | - | - | ns | ns | ns |
|  | *R*_d_ | - | - | - | ns | <0.05 | ns |
|  |  |  |  |  |  |  |  |

**Supporting Information Table S2**. Energy content of soybean vegetative and reproductive organs. Pod+seed energy was measured and compared between WT and light green *Y11y11* from beginning podfill (early R5) to maturity (R8). Values from Amthor *et al*. (1994) are included for comparison. Different letters represent significant differences at alpha=0.1.

|  | Energy content | | |
| --- | --- | --- | --- |
|  | (MJ kg^-1^) | | |
| Plant tissue | WT | *Y11y11* | Amthor *et al*. (1994) |
| Leaf | - | - | 19.0 |
| Stem | - | - | 17.2 |
| Pod+seed (early R5) | 17.1a | 17.2a | - |
| Pod+seed (late R5) | 18.9a | 17.9b | - |
| Pod+seed (R6) | 20.8a | 20.6a | - |
| Pod+seed (R8) | 21.7a | 21.6a | 22.8 |
| Seed (R8) | 23.2a | 23.1a | - |


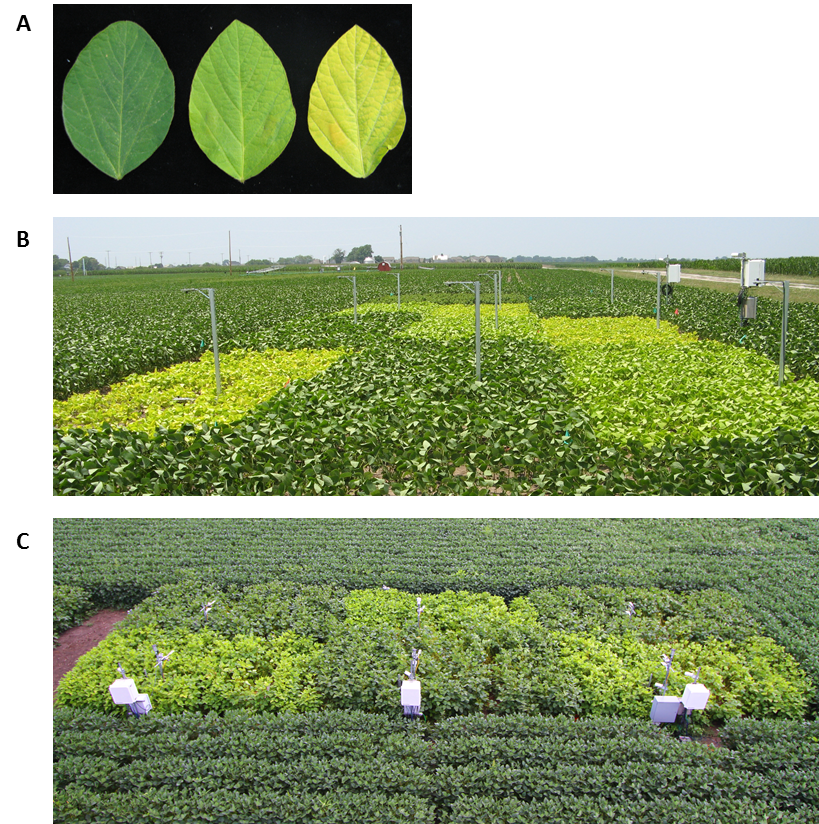


**Supporting Information Figure S1**. Phenotypes and experimental design of dark- and light-green soybean. A) Leaves of WT ‘Clark’ (dark green, left), *Y11y11* (light green, center), and *y9y9* (yellow/green, right) plants show the difference in chl content early in the 2012 season. B) All three genotypes were grown in the field in 2012 using the 0.38 m row spacing. C) Only WT and *Y11y11* were grown in the 2013 season using the 0.38 m row spacing (shown above) and the narrower 0.19 m row spacing in the same design (not shown). All experiments had a sample size of n=3.


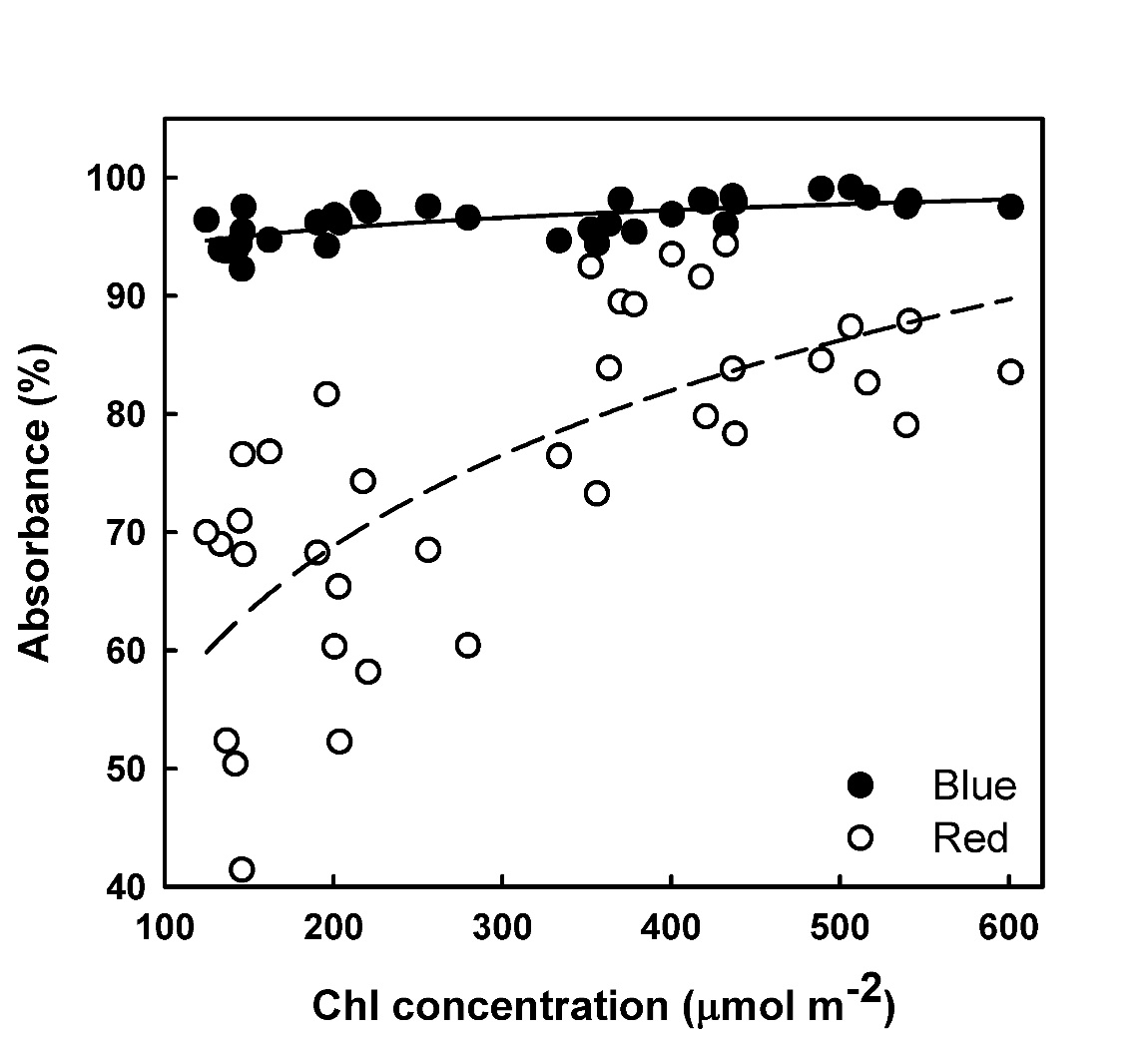


**Supporting Information Figure S2**. Relationships between chl concentration and absorbance in the blue and red wavelengths emitted by the gas exchange system diodes (LI-6400, LI-COR, Lincoln, NE, USA). Relationships were used to calculate leaf absorbance in 2012. Solid line: y=2.207ln(x)+84.014 (r^2^=0.42); dashed line: y=19.007ln(x)-31.879 (r^2^=0.51)


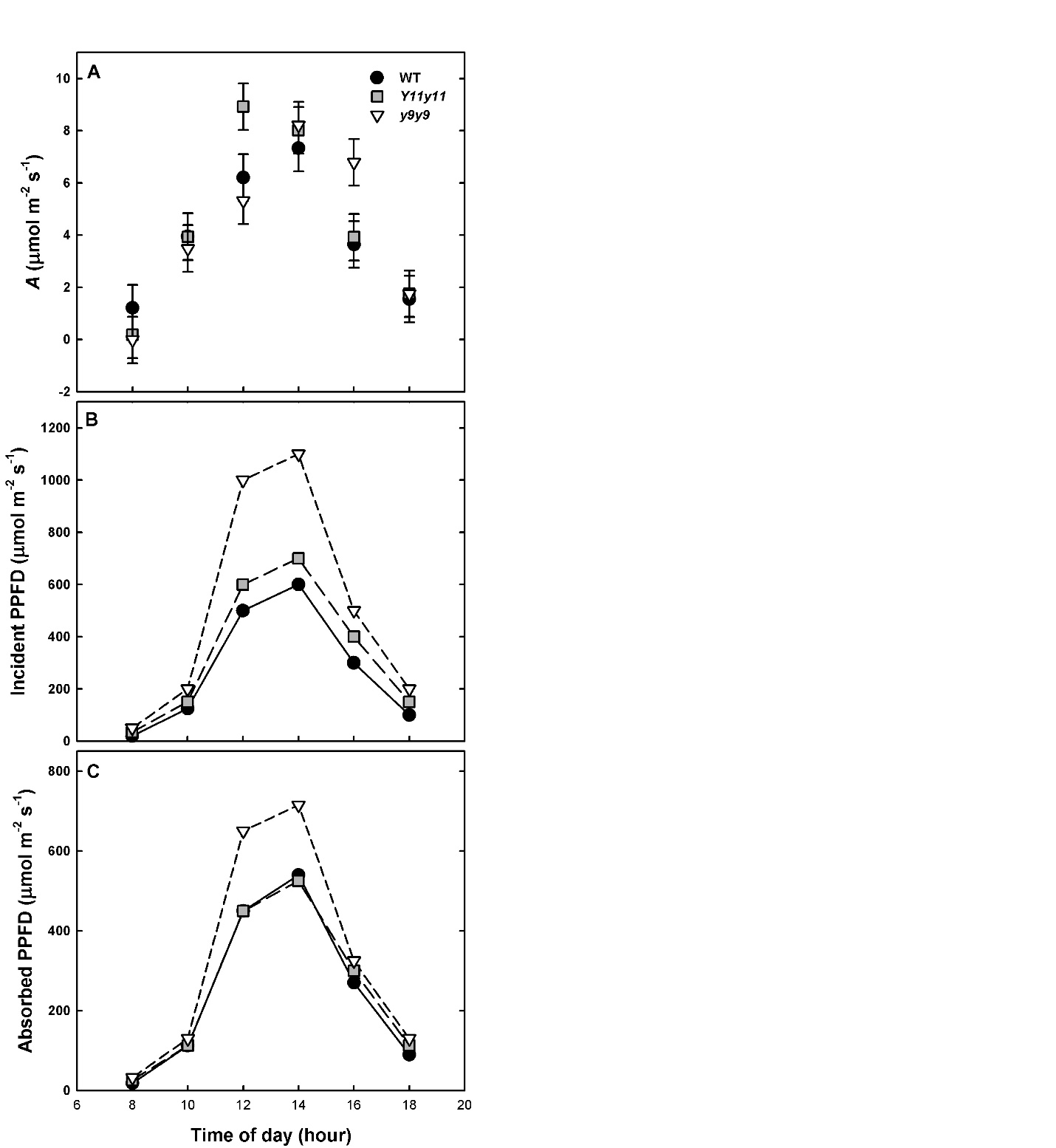


**Supporting Information Figure S3.** Diurnal gas exchange measurements in shade leaves during the 2012 season. Shade *A*_leaf_ (A) was measured using an open path gas analyzer at six points during DOY 191 in WT dark green soybean (black circles) and two chl-deficient soybean mutants (*Y11y11*=gray squares; *y9y9*=white triangles). Incident PPFD (B) was measured using a line quantum sensor and averaged over all blocks. Using a relationship between chl content and absorbance of the wavelengths used by the open path gas analyzer, absorbed PPFD (C) was calculated. Error bars represent the stand errors (n=3).


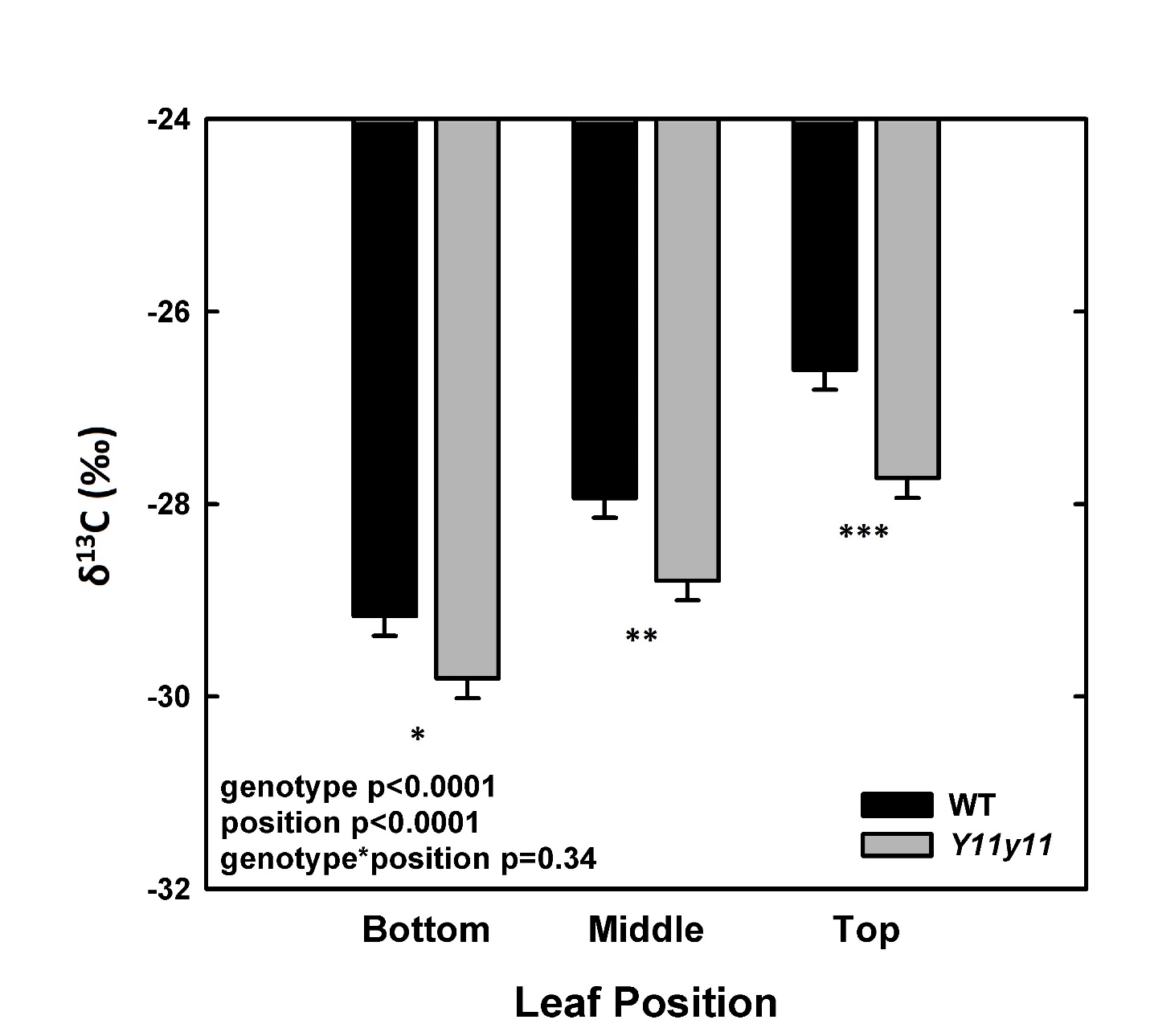


**Supporting Information Figure S4**. Isotopic measurements of leaf δ^13^C in WT (black) and *Y11y11* (gray) at three positions in the canopy. Error bars represent the standard error (n=3). Within-DOY differences are indicated by *p<0.05, **p<0.01, ***p<0.001.


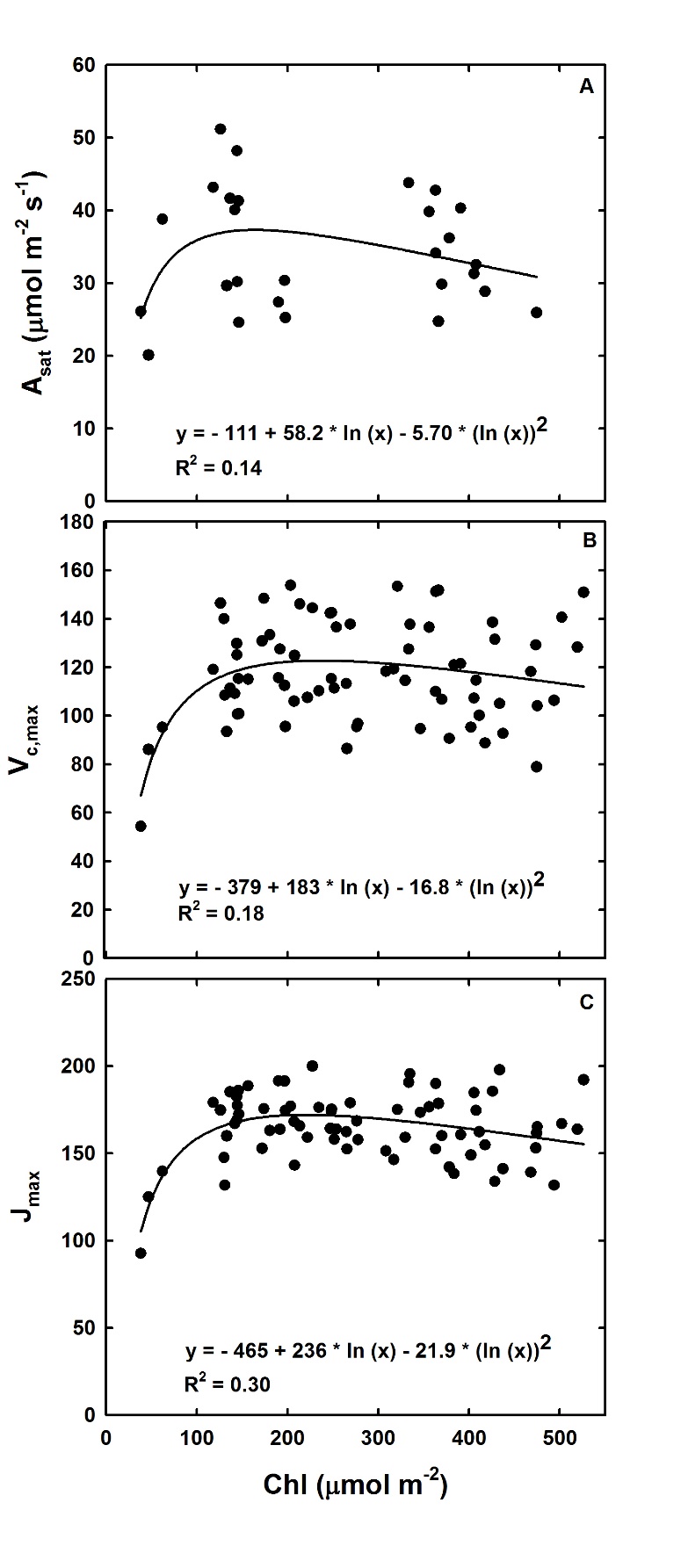


**Supporting Information Figure S5.** Relationships between photosynthetic parameters and chl content. The maximum rate of photosynthesis (*A*_sat_, A), maximum carboxylation rate of Rubisco (*V*_c,max_, B), and maximum electron transport rate (*J*_max_, C) are plotted as a function of chl content across all genotypes (WT, *Y11y11*, *y9y9*) and years (2012, 2013) using data from experimental units, as opposed to overall means. Functions of best fit are indicated, in which all parameters were significant at alpha=0.1.


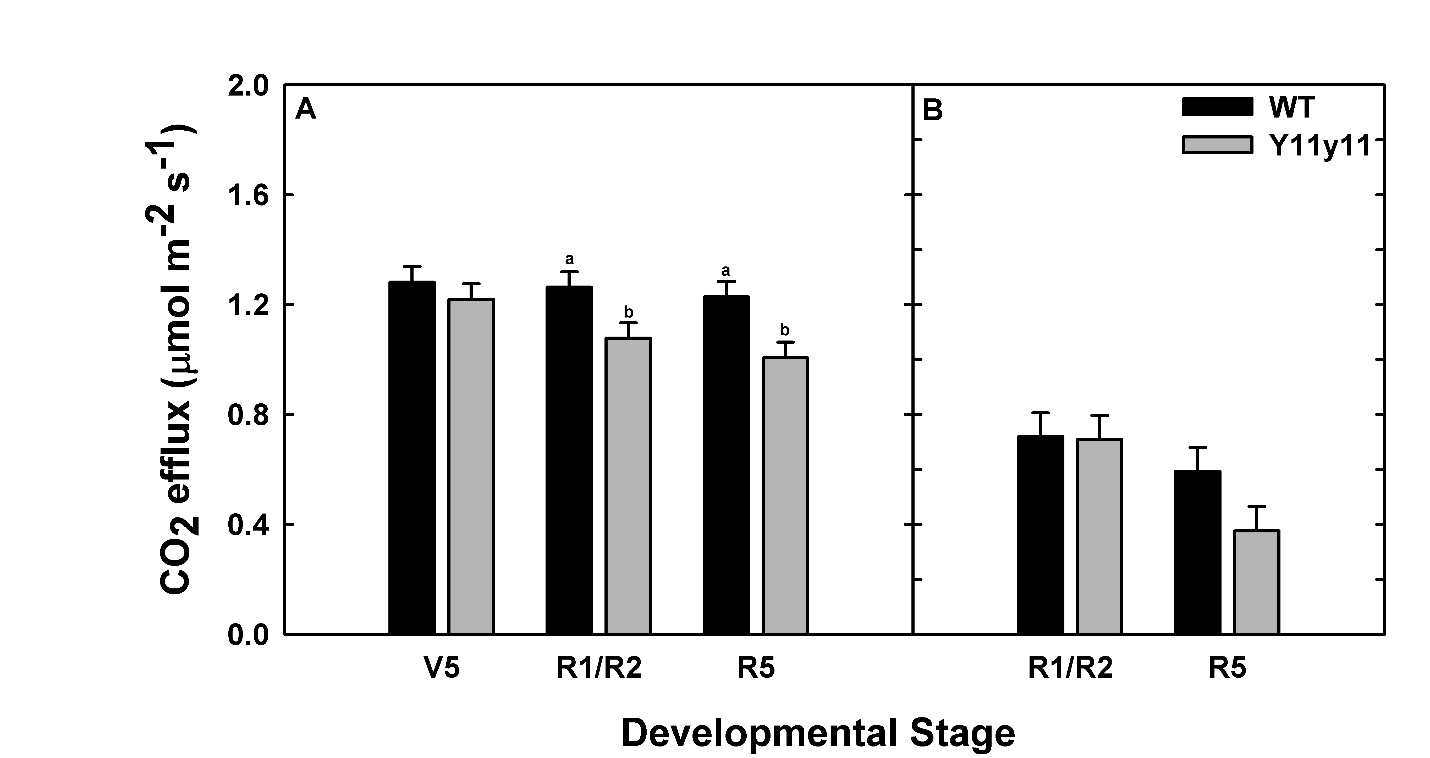


**Supporting Information Figure S6**. Respiration rates in 2013 across developmental stage in two leaf layers. Mean respiration (CO_2_ efflux) is shown for WT (black) and *Y11y11* (gray) sun (A) and shade (B) leaves. Sun leaves were measured in V5, R1/R2, and R5 whereas shade leaves were only measured after canopy closure (R1/R2 and R5). Error bars represent the standard error (n=3). Letters represent significant differences within developmental stage (alpha=0.1) when present.
